# Association of Subcutaneous or Intravenous Administration of Casirivimab and Imdevimab Monoclonal Antibodies With Clinical Outcomes in Adults With COVID-19

**DOI:** 10.1001/jamanetworkopen.2022.6920

**Published:** 2022-04-12

**Authors:** Erin K. McCreary, J. Ryan Bariola, Richard J. Wadas, Judith A. Shovel, Mary Kay Wisniewski, Michelle Adam, Debbie Albin, Tami Minnier, Mark Schmidhofer, Russell Meyers, Oscar C. Marroquin, Kevin Collins, William Garrard, Lindsay R. Berry, Scott Berry, Amy M. Crawford, Anna McGlothlin, Kelsey Linstrum, Anna Nakayama, Stephanie K. Montgomery, Graham M. Snyder, Donald M. Yealy, Derek C. Angus, Paula L. Kip, Christopher W. Seymour, David T. Huang, Kevin E. Kip

**Affiliations:** 1 Division of Infectious Diseases, Department of Medicine, University of Pittsburgh School of Medicine, Pittsburgh, Pennsylvania; 2 Department of Emergency Medicine, University of Pittsburgh School of Medicine, Pittsburgh, Pennsylvania; 3 Wolff Center, University of Pittsburgh Medical Center (UPMC), Pittsburgh, Pennsylvania; 4 Supply Chain Management/HC Pharmacy, UPMC, Pittsburgh, Pennsylvania; 5 Division of Cardiology, Department of Medicine, University of Pittsburgh School of Medicine, Pittsburgh, Pennsylvania; 6 Clinical Analytics, UPMC, Pittsburgh, Pennsylvania; 7 Berry Consultants, Austin, Texas; 8 Office of Healthcare Innovation, UPMC, Pittsburgh, Pennsylvania; 9 Clinical Research Investigation and Systems Modeling of Acute Illness (CRISMA) Center, Department of Critical Care Medicine, University of Pittsburgh School of Medicine, Pittsburgh, Pennsylvania; 10 Department of Critical Care Medicine, University of Pittsburgh School of Medicine, Pittsburgh, Pennsylvania

## Abstract

**Importance:**

Monoclonal antibody (mAb) treatment decreases hospitalization and death in high-risk outpatients with mild to moderate COVID-19; however, only intravenous administration has been evaluated in randomized clinical trials of treatment. Subcutaneous administration may expand outpatient treatment capacity and qualified staff available to administer treatment, but the association with patient outcomes is understudied.

**Objectives:**

To evaluate whether subcutaneous casirivimab and imdevimab treatment is associated with reduced 28-day hospitalization and death compared with nontreatment among mAb-eligible patients and whether subcutaneous casirivimab and imdevimab treatment is clinically and statistically similar to intravenous casirivimab and imdevimab treatment.

**Design, Setting, and Participants:**

This prospective cohort study evaluated high-risk outpatients in a learning health system in the US with mild to moderate COVID-19 symptoms from July 14 to October 26, 2021, who were eligible for mAb treatment under emergency use authorization. A nontreated control group of eligible patients was also studied.

**Exposures:**

Subcutaneous injection or intravenous administration of the combined single dose of 600 mg of casirivimab and 600 mg of imdevimab.

**Main Outcomes and Measures:**

The primary outcome was the 28-day adjusted risk ratio or adjusted risk difference for hospitalization or death. Secondary outcomes included 28-day adjusted risk ratios and differences in hospitalization, death, a composite end point of emergency department admission and hospitalization, and rates of adverse events. Among 1959 matched adults with mild to moderate COVID-19, 969 patients (mean [SD] age, 53.8 [16.7] years; 547 women [56.4%]) who received casirivimab and imdevimab subcutaneously had a 28-day rate of hospitalization or death of 3.4% (22 of 653 patients) compared with 7.0% (92 of 1306 patients) in nontreated controls (risk ratio, 0.48; 95% CI, 0.30-0.80; *P* = .002). Among 2185 patients treated with subcutaneous (n = 969) or intravenous (n = 1216; mean [SD] age, 54.3 [16.6] years; 672 women [54.4%]) casirivimab and imdevimab, the 28-day rate of hospitalization or death was 2.8% vs 1.7%, which resulted in an adjusted risk difference of 1.5% (95% CI, −0.6% to 3.5%; *P* = .16). Among all infusion patients, there was no difference in intensive care unit admission (adjusted risk difference, 0.7%; 95% CI, −3.5% to 5.0%) or need for mechanical ventilation (adjusted risk difference, 0.2%; 95% CI, −5.8% to 5.5%).

**Conclusions and Relevance:**

In this cohort study of high-risk outpatients with mild to moderate COVID-19 symptoms, subcutaneously administered casirivimab and imdevimab was associated with reduced hospitalization and death when compared with no treatment. These results provide preliminary evidence of potential expanded use of subcutaneous mAb treatment, particularly in areas that are facing treatment capacity and/or staffing shortages.

## Key Points

**Question:**

Is subcutaneous treatment with casirivimab and imdevimab associated with improved 28-day clinical outcomes compared with nontreatment, and is it clinically similar to intravenously administered casirivimab and imdevimab for outpatients with COVID-19?

**Findings:**

In this cohort study of 1959 propensity-matched outpatients with mild to moderate COVID-19 symptoms, the 28-day rate of hospitalization or death was 3.4% vs 7.0% for those receiving subcutaneous treatment vs nontreatment. In a second cohort analysis of 2185 outpatients, the 28-day rate of hospitalization or death was 2.8% vs 1.7% for subcutaneous vs intravenous treatment.

**Meaning:**

Subcutaneous casirivimab and imdevimab was associated with reduced hospitalization and death compared with nontreatment and showed similar outcomes compared with intravenous casirivimab and imdevimab in outpatients with COVID-19.

## Teaser

This cohort study evaluates the clinical outcomes of patients with COVID-19 receiving subcutaneous vs intravenous treatment with casirivimab and imdevimab.

## Introduction

Discovery and broadscale implementation of therapies that decrease progression to severe COVID-19 and improve mortality of patients infected with SARS-CoV-2 are critical for global health. Casirivimab and imdevimab are monoclonal antibodies (mAbs) that decrease hospitalizations and death in high-risk outpatients with mild to moderate COVID-19 when used as treatment and decrease symptomatic COVID-19 when used as postexposure prophylaxis.^1,2^ These agents are available under emergency use authorization (EUA) for these indications in the US, UK, and other global communities.^3,4^ Only intravenous administration was evaluated in randomized clinical trials for treatment, and, accordingly, intravenous infusion is strongly recommended per the US Food and Drug Administration for this indication. However, the EUA states that subcutaneous injection is an alternative route of administration when intravenous infusion is not feasible and would lead to delay in treatment, although the efficacy of subcutaneous injection for treatment of SARS-CoV-2 is unknown.

A COVID-19 surge in September 2021, coupled with health care worker staffing shortages, resulted in a capacity crisis for outpatient mAb infusions at our learning health system. Key stakeholders and clinical leaders determined that continuation of intravenous therapy would delay or prevent treatment for mAb referrals, and conversion to subcutaneous injections would add treatment capacity, reduce appointment times, and expand staff available to administer treatment. The purpose of this study was to evaluate whether subcutaneous casirivimab and imdevimab treatment is associated with reduced risk-adjusted 28-day clinical outcomes compared with nontreatment with mAb. We also sought to evaluate the similarity of clinical outcomes comparing subcutaneous with intravenous treatment to inform future operations within our learning health system.

## Methods

This prospective cohort study evaluated patients within the Optimizing Treatment and Impact of Monoclonal Antibodies Through Evaluation for COVID-19 embedded learning platform.^5^ This study was approved by the University of Pittsburgh Medical Center (UPMC) Quality Improvement Review Committee and the University of Pittsburgh institutional review board as an exempt protocol. Patients verbally consented to mAb treatment and reviewed the US Food and Drug Administration EUA fact sheet before treatment. Data were deidentified for this analysis. Methods and results are reported in accordance with the Reporting of Studies Conducted Using Observational Routinely Collected Health Data (RECORD) statement.^16^ The study followed the Strengthening the Reporting of Observational Studies in Epidemiology (STROBE) reporting guideline.

From platform launch on March 10, 2021, through September 9, 2021, all patients were assigned intravenous mAb treatment via a central management system. A few patients received casirivimab and imdevimab subcutaneously if they presented directly to an urgent care facility within the system. From September 9 through October 26, 2021, most outpatient infusion centers provided only subcutaneous injections of casirivimab and imdevimab to accommodate surging patient referrals and staffing shortages because continuing intravenous treatment only would lead to a delay in care (per EUA language). After October 26, 2021, centers converted back to intravenous administration when feasible within workforce capacity as case volumes decreased and intravenous treatment no longer resulted in care delay. Starting on September 28, 2021, patients 65 years or older with loss of 2 or more activities of daily living, pregnant patients, and/or patients with immunocompromised conditions were given priority for mAb treatment appointment scheduling.

Adverse events (defined as any reaction that occurred during injection or in the observation period after injection, eg, rash, shortness of breath, or hypertension) were recorded by practitioners at each infusion center in a secure electronic file–sharing application. Nursing and physician staff also used an internal, nonpunitive, patient safety reporting system (Risk Master) for adverse reactions and medication errors. Data from these 2 sources were combined and deidentified. A blinded attending physician trained in emergency medicine (R.J.W.) reviewed the deidentified data to determine severity. An infusion reaction management guide was created and distributed to all mAb treatment sites for guidance on treatment of any kind of mAb-related adverse event (eTable 1 in the Supplement).

### Outcomes

For this analysis, the 2 research questions were (1) whether subcutaneous casirivimab and imdevimab treatment is associated with better 28-day clinical outcomes than nontreatment among mAb-eligible patients and (2) whether subcutaneous casirivimab and imdevimab treatment is clinically and statistically similar to intravenous casirivimab and imdevimab treatment. The primary outcome was the 28-day adjusted risk ratio of hospitalization or death for question 1, and the 28-day adjusted risk difference of hospitalization/death for question 2. Secondary outcomes included 28-day adjusted risk ratios and differences of hospitalization, death, a composite end point of emergency department (ED) admission and hospitalization, and rates of adverse events. In post hoc analyses, the risk of severe hospitalization, defined as an intensive care unit (ICU) admission or mechanical ventilation, was examined. Adverse events were defined as above. Death was ascertained by the code “ceased to breathe” in the electronic medical record as well as supplemented with deaths identified by the Social Security Administration Death Master File, which is received monthly by UPMC. Ventilation was based on Healthcare Common Procedure Coding System code 94002 or 94003. Hospitalization, ED admission, and ICU admission were based on admission charge data from each treating facility.

### Selection of Patient Analysis Cohorts

For the first research question, nontreated control participants were selected from nonhospitalized patients 12 years or older who had a positive SARS-CoV-2 polymerase chain reaction or antigen test result within our health care system from July 14 to October 26, 2021. These patients, whose symptom status was unknown, had an EUA-eligible risk factor for progression to severe disease and no admission to the emergency department or hospital on the date of their positive SARS-CoV-2 test result (ie, presumed not to be at imminent risk of hospitalization). July 14, 2021, was chosen as the start date for this analysis because this was the first confirmed date, per national tracking data, that 100% of patients infected with COVID-19 in our system had the Delta variant; Delta remained the only regional variant until the end of the study period.^6,7^ Corresponding treated individuals were patients 12 years or older treated subcutaneously with casirivimab and imdevimab in an outpatient infusion center or urgent care facility during the same period as nontreated control participants. Patients who received mAb treatment in the emergency department were excluded because the subcutaneous route of administration was not used in that setting. Both groups required a 28-day follow-up period. For nontreated control participants, the 28-day outcome ascertainment period started on the day after the positive SARS-CoV-2 test result. For treated patients, the 28-day outcome ascertainment period started on the day of mAb treatment.

For the second research question, patients treated subcutaneously or intravenously at an outpatient infusion center or urgent care facility on or after July 14, 2021, and with an available follow-up period of 28 days were compared. For both groups, the 28-day outcome ascertainment period started on the day of treatment. Because not all clinical sites provided subcutaneous mAb treatment, separate study populations were compiled, including all mAb-treated patients and the subset of mAb-treated patients at clinical sites in which both routes of administration were used (ie, to remove a potential site effect from the larger analysis). For this cohort to simply examine a scheduled change in practice, patients receiving treatment intravenously were treated from July 15 to September 8, 2021, and patients receiving treatment subcutaneously were treated from September 9 to September 29, 2021 (ie, nonoverlapping treatment periods).

### Data Sources

We used health-related data captured in the electronic health record and ancillary clinical systems, all of which are aggregated and harmonized in a clinical data warehouse.^8,9^ For infusion sites with complete electronic medical record data in the clinical data warehouse, we accessed sociodemographic data, medical history, and billing charges for all outpatient and in-hospital encounters with diagnoses and procedures coded based on the *International Classification of Diseases, Ninth Revision* and *International Statistical Classification of Diseases and Related Health Problems, Tenth Revision* (eTable 2 in the Supplement).^10,11^ Race was classified as Black, White, or other (including Alaska Native, American Indian, Asian, Filipino, Indian, Native Hawaiian, or Pacific Islander). We assessed 28-day mortality by the hospital discharge disposition of “ceased to breathe” sourced from the inpatient medical record system, as well as deaths after discharge identified with the Death Master File from the Social Security Administration^13^ and the 2021 National Technical Information Service as an external data source.^12^

### Statistical Analysis

Sociodemographic and clinical characteristics were compared between patients treated subcutaneously and nontreated control participants by use of standardized mean differences. To control for imbalances in patient profiles between the 2 groups, we selected nontreated control participants matched to treated patients by the propensity score method.^14,15^ Specifically, propensity scores were derived from a logistic regression model fit with subcutaneous mAb treatment as the response variable and selection of measured pretreatment explanatory variables (eTable 3 in the Supplement) based on (1) presumed biological relevance (age, sex, and race), (2) standardized mean difference of 0.10 or greater between treated and nontreated participants, and (3) other selection criteria (eTable 4 in the Supplement). Missing values for covariates were not imputed in this analysis; thus, final sample sizes for analysis reflect patients with complete data for all variables included in the propensity score.

We used 1:2 propensity score nearest-neighbor matching with a maximum propensity score probability difference of 0.01 to construct the matched treated and nontreated groups. We performed nonmatched parallel analyses in which outcomes of treated participants were compared with nontreated participants using (adjusting for) the propensity score as a covariate and with inverse probability weighting. Both the matched and nonmatched adjusted analyses were conducted using generalized linear models with mAb receipt as the variable of interest, specifying the binomial distribution and log link. We did not impute missing values for variables used in deriving the propensity scores.

Sociodemographic and clinical characteristics were also compared between participants treated subcutaneously and intravenously by use of 2-tailed, unpaired *t* tests or Wilcoxon rank sum tests for continuous variables and χ^2^ tests for categorical variables. Because treatment regimen was selected based on logistical (supply) constraints rather than clinical considerations, we identified any unanticipated between-group imbalances of baseline characteristics using *P* < .05. We then fit a logistic regression model with age, sex, Black race, and vaccination status included based on presumed relevance (advanced age, male sex, Black race, and unvaccinated patients have worse outcomes from COVID-19) and used forward stepwise selection. No additional variables were added to the model. Given low event rates and to preserve the full sample, Black race (1.7% missing) was coded as a value of 1 for known Black race, and vaccination status (12.8% missing) was coded as 1 when full vaccination status was known. The primary parameter of interest was the adjusted risk difference (subcutaneous − intravenous) in the 28-day rate of hospitalization or death, with a boundary of 3% used to define similar clinical outcome. The 3% boundary was decided a priori as a consensus threshold that was clinically meaningful for the health care system population and for capacity management. All analyses were performed using SAS statistical software, version 9.4 (SAS Institute Inc).

## Results

### Study Populations

**Figure 1.**
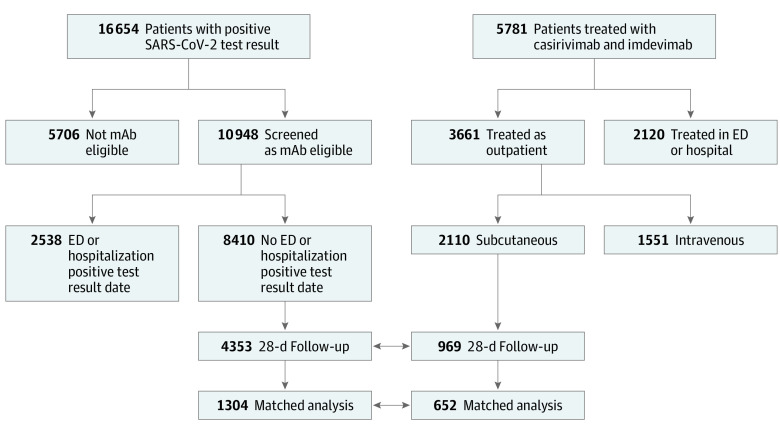
Flow Diagram of the Selection Criteria and Patient Populations Used in the Analysis of Subcutaneous Treatment With Casirivimab and Imdevimab and Nontreated Control Participants ED indicates emergency department; mAb, monoclonal antibody.

**Figure 2.**
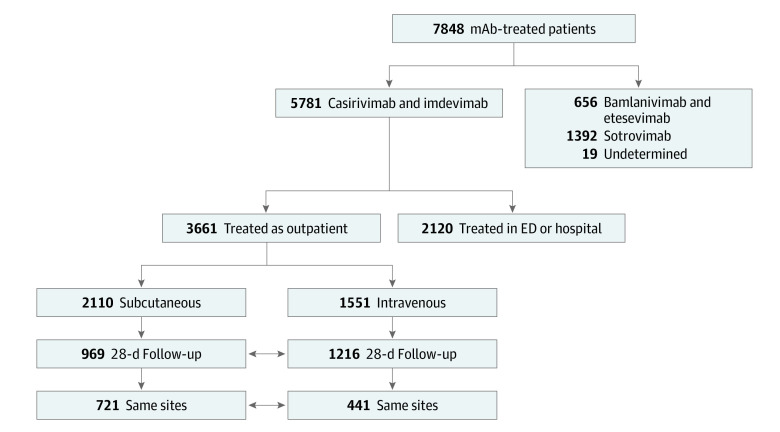
CONSORT Diagram of the Selection Criteria and Patient Populations Used in the Analysis of Subcutaneous and Intravenous Treatment ED indicates emergency department; mAb, monoclonal antibody.

A total of 1959 matched adults with mild to moderate COVID-19 participated in this cohort study. The first analysis included 969 patients treated subcutaneously with casirivimab and imdevimab (mean [SD] age, 53.8 [16.7] years; 547 female [56.4%] and 422 male [42.6%]; and 49 Black [5.2%], 852 White [89.8%], and 48 of other race or ethnicity [5.1%]) and 4353 nontreated, EUA-eligible controls in the unmatched cohort (mean [SD] age, 49.7 [21.5] years; 2542 female [58.4%]; and 407 Black [9.5%], 3824 White [88.8%], and 73 of other race or ethnicity [1.7%]). The propensity score–matched analysis, which required complete covariate data and matching (as defined in the Methods), compared 652 patients treated subcutaneously with casirivimab and imdevimab with 1304 nontreated, EUA-eligible controls (Figure 1). For the second analysis, the 969 subcutaneously treated patients were compared with 1216 patients (mean [SD] age, 54.3 [16.6] years; 672 female [54.4%] and 554 male [45.6%]; and 83 Black [6.9%], 1071 White [89.4%], and 44 of other race or ethnicity [3.7%]) treated with the same mAb intravenously. From this cohort, 721 patients treated subcutaneously vs 441 treated intravenously were treated at clinical sites in which both routes of administration were used during the study period (Figure 2).

### Matched Analysis of Treated and Nontreated Patients

Before matching on propensity score (based on covariate data), subcutaneously treated patients (who were selected using a priority system that favored older age and being immunocompromised starting on September 28, 2021) were older and less likely of Black race than nontreated patients (eTable 4 in the Supplement). In addition, treated patients had a higher prevalence of rheumatoid arthritis, obstructive sleep apnea, hypertension, and smoking history than nontreated patients. This overall higher risk profile of treated patients was also reflected in a higher prevalence of statin and β-blocker use than nontreated patients. Of importance, after propensity score matching, treated and nontreated patients were similar (standardized mean difference, <0.10) on variables included in the propensity score model (eTable 4 in the Supplement), the distribution of propensity scores (eFigure 1 in the Supplement), and variables not included in the model (eTable 4 in the Supplement).

The matched 28-day rate of hospitalization or death was 3.4% in treated patients compared with 7.0% in nontreated controls (Table 1). The corresponding risk ratio for 28-day hospitalization or death was 0.48 (95% CI, 0.30-0.80; *P* = .002). The lower risk of hospitalization or death in treated patients was most evident in the first 15 days of follow-up (eFigure 2 in the Supplement). The 28-day death rate was 0.2% in the treated group vs 2.4% in the nontreated group (*P* = .007 from the log-binomial regression model).

**Table 1.** Propensity Score–Matched 28-Day Event-Free Rates and Risk Ratios of Study Outcomes

Outcome	No. (%) of events		Risk ratio (95% CI)	*P* value
	Treated (n = 653)	Nontreated (n = 1306)		
Hospitalization or death	22 (3.4)	92 (7.0)	0.48 (0.3-0.8)	.002
Hospitalization	22 (3.4)	72 (5.5)	0.61 (0.4-1.0)	.04
Death	1 (0.2)	31 (2.4)	0.06 (0.0-0.5)	.007
ED admission or hospitalization	40 (6.1)	129 (9.9)	0.62 (0.4-0.9)	.006

Abbreviation: ED, emergency department.

### Unmatched Analysis of Treated and Nontreated Patients

In unmatched patients with a propensity score (ie, covariate data), the crude 28-day rate of hospitalization or death was 3.5% in treated patients compared with 6.6% in nontreated controls (eTable 2 in the Supplement). The corresponding risk ratio for hospitalization or death adjusted for the propensity score was 0.46 (95% CI, 0.30-0.70; *P* < .001). Results were relatively consistent with the use of inverse probability weighting. As in the matched analysis, deaths were infrequent, with the 28-day death rate being lower in the treated group (0.2%) compared with the nontreated group (2.1%) (adjusted risk ratio, 0.06; 95% CI, 0.01-0.44; *P* = .005).

### Sensitivity Analysis

Information on symptoms for untreated controls was not available on the date of their positive COVID-19 test result. For treated patients with a positive test result documented in the UPMC system, the median time from symptom onset to the positive test date was 3 days (IQR, 2-5 days), and the median time from positive test date to infusion was 2 days (IQR, 2-4 days). Therefore, to assess potential immortal time bias, we modified the untreated control group follow-up period to start 2 or 3 days after the positive COVID-19 test result date. The corresponding propensity score–adjusted risk ratios (treated vs untreated) for 28-day hospitalization or death were 0.56 (95% CI, 0.36-0.85) for 2 days and 0.63 (95% CI, 0.41-0.97) for 3 days.

### Evaluation of Subcutaneous and Intravenous Treatment

Patients treated subcutaneously or intravenously had generally similar demographic and presenting clinical characteristics (Table 2). This overall similarity in patient profiles was evident among all treated patients as well as the subset of patients treated at clinical sites in which both routes of mAb administration were used. A notable exception was a higher rate of full COVID-19 vaccination in patients treated subcutaneously (447 [55.5%]) compared with those treated intravenously (485 [44.1%]) in patients from all sites (*P* < .001); however, this rate was similar in patients treated within the same sites. The mean (SD) time from symptom onset to infusion was 6.1 (1.9 or 2.0) days in both groups.

**Table 2.** Comparison of Characteristics of Patients Treated With Subcutaneous and Intravenous Monoclonal Antibodies^a^

Characteristic	All patients receiving infusions^b^			Patients receiving infusions at the same site^c^		
	Subcutaneous (n = 969)	Intravenous (n = 1216)	*P* value	Subcutaneous (n = 721)	Intravenous (n = 441)	*P* value
Age, mean (SD), y	53.8 (16.7)	54.3 (16.6)	.45	54.5 (16.5)	53.9 (17.4)	.57
Sex						
Female	547 (56.4)	672 (54.4)	.35	401 (55.6)	227 (51.5)	.17
Male	422 (42.6)	554 (45.6)		320 (44.4)	214 (48.5)	
Race and ethnicity						
Black	49 (5.2)	83 (6.9)	.08	32 (4.5)	24 (5.5)	.55
White	852 (89.8)	1071 (89.4)		640 (89.1)	392 (89.3)	
Other^d^	48 (5.1)	44 (3.7)		46 (6.4)	23 (5.25)	
BMI, mean (SD)	31.8 (7.5)	32.8 (8.4)	.03	32.0 (7.8)	32.3 (8.4)	.62
History						
Smoking	227 (34.0)	135 (31.2)	.35	171 (33.9)	113 (31.0)	.46
Diabetes	112 (16.8)	161 (17.1)	.86	93 (18.4)	58 (16.2)	.39
Obstructive sleep apnea	128 (19.2)	174 (18.5)	.73	106 (21.0)	76 (21.2)	.95
Dyspnea	40 (6.0)	69 (7.3)	.29	33 (6.5)	17 (4.7)	.26
Asthma	220 (32.9)	283 (30.0)	.22	164 (32.5)	114 (31.7)	.82
Pulmonary hypertension	13 (1.9)	9 (1.0)	.09	11 (2.2)	1 (0.3)	.02
COPD	115 (17.2)	151 (16.0)	.53	84 (16.6)	54 (15.0)	.53
Hypertension	314 (47.0)	408 (43.3)	.14	253 (50.1)	158 (44.0)	.08
Atrial fibrillation	33 (4.9)	55 (5.8)	.43	27 (5.4)	20 (5.6)	.87
Valvular heart disease	31 (4.6)	68 (7.2)	.03	26 (5.2)	29 (8.1)	.08
Coronary artery disease	73 (10.9)	105 (11.1)	.89	61 (12.1)	45 (12.5)	.84
Stroke	35 (5.2)	37 (3.9)	.21	28 (5.5)	16 (4.5)	.47
Congestive heart failure	36 (5.4)	50 (5.3)	.94	26 (5.1)	13 (3.6)	.29
Chronic kidney disease	34 (5.1)	63 (6.7)	.18	24 (4.7)	24 (6.7)	.22
Fatty liver disease	28 (4.2)	23 (2.4)	.05	23 (4.5)	11 (3.1)	.27
Cancer	70 (10.5)	127 (13.5)	.07	53 (10.5)	57 (15.9)	.02
Chemotherapy	31 (4.6)	35 (3.7)	.36	26 (5.1)	26 (7.2)	.20
Allergic rhinitis	78 (11.7)	140 (14.9)	.07	56 (11.1)	47 (13.1)	.37
Rheumatoid arthritis	24 (3.6)	26 (2.8)	.34	17 (3.4)	15 (4.2)	.53
Viral hepatitis	12 (1.8)	10 (1.1)	.21	5 (1.0)	2 (0.6)	.48
Solid organ or cell transplant	10 (1.5)	10 (1.1)	.44	4 (0.8)	7 (1.9)	.22
Drug class						
ACE inhibitors	118 (17.7)	138 (14.7)	.10	105 (20.8)	52 (14.5)	.02
Angiotensin II receptor blockers	69 (10.3)	102 (10.8)	.75	53 (10.5)	45 (12.5)	.35
α-Blockers	9 (1.3)	7 (0.7)	.23	6 (1.2)	1 (0.3)	.14
β-Blockers	166 (24.8)	198 (21.0)	.07	126 (24.9)	89 (24.8)	.96
Statins	223 (33.4)	333 (35.4)	.41	181 (35.8)	134 (37.3)	.66
Antidepressants	200 (29.9)	314 (33.3)	.15	153 (30.3)	132 (36.8)	.05
Corticosteroids as a home medication	240 (35.9)	340 (36.1)	.95	181 (35.8)	114 (31.8)	.21
Charlson Comorbidity Index score	0.8 (1.4)	0.8 (1.3)	.95	0.8 (1.4)	0.8 (1.2)	.75
Time from symptoms to infusion, mean (SD), d	6.1 (1.9)	6.1 (2.0)	.95	6.1 (1.9)	6.1 (1.9)	.83
Symptoms to infusion group						
1-4 d	170 (21.2)	257 (23.4)	.11	144 (21.0)	85 (20.9)	.73
5-6 d	293 (36.5)	352 (32.0)		248 (36.3)	139 (34.1)	
≥7 d	339 (42.3)	491 (44.6)		292 (42.7)	183 (45.0)	
Vaccination status						
Unvaccinated	267 (33.2)	455 (41.4)	<.001	232 (33.8)	141 (34.6)	.75
Partially vaccinated	22 (2.7)	26 (2.4)		18 (2.6)	14 (3.4)	
Fully vaccinated^e^	447 (55.5)	485 (44.1)		372 (54.2)	210 (51.6)	
Unknown or not determined	69 (8.6)	134 (12.2)		64 (9.3)	42 (10.3)	

Abbreviations: ACE, angiotensin-converting enzyme; BMI, body mass index (calculated as weight in kilograms divided by height in meters squared); COPD, chronic obstructive pulmonary disease.

^a^ Data are presented as number (percentage) of study participants unless otherwise indicated.

^b^ Patients treated at all health system facilities. Patients were treated subcutaneously from July 20 to September 29, 2021. Patients were treated intravenously from July 15 to September 29, 2021.

^c^ Patients treated at the same health system facilities. Patients were treated subcutaneously from September 9 to 29, 2021. Patients were treated intravenously from July 15 to September 8, 2021.

^d^ Other race (self-reported) included Alaska Native, American Indian, Asian, Filipino, Indian, Native Hawaiian, and Pacific Islander.

^e^ Defined as at least 2 doses of the Pfizer or Moderna vaccine or at least 1 dose of the Johnson & Johnson vaccine.

For all patients receiving infusions, the adjusted risk difference of hospitalization or death comparing patients treated subcutaneously and intravenously was 1.5% (95% CI, −0.6% to 3.5%; *P* = .16), which was within the clinically predefined similarity boundary of 3%, yet the upper limit of the 95% CI exceeded this boundary (Table 3). The corresponding adjusted risk ratio was 1.71 (95% CI, 0.97-3.00; *P* = .06). For hospitalization, the adjusted risk ratio was 1.79 (95% CI, 1.01-3.17; *P* = .05). Adjusted risk differences of death and ED admission or hospitalization were small (similar) by route of administration. In terms of initial safety, rates of severe adverse reactions were 0.0% in patients receiving subcutaneous treatment and 0.2% in patients receiving intravenous treatment.

**Table 3.** 28-Day Risk Differences and Risk Ratios of Hospitalization or Death by Route of Monoclonal Antibody Administration^a^

Outcome	No. (%) of patients		Absolute risk difference, %			Adjusted risk ratio^a^	
	Subcutaneous (969 total patients and 721 infused at same site)	Intravenous (1216 total patients and 441 infused at same site)	Unadjusted	Adjusted^b^	95% CI	Adjusted (95% CI)	*P* value
All patients receiving infusions							
Hospitalization or death	27 (2.8)	21 (1.7)	1.1	1.5	−0.6 to 3.5	1.7 (1.0 to 3.0)	.06
Hospitalization	27 (2.8)	20 (1.6)	1.1	1.5	−0.4 to 3.4	1.8 (1.0 to 3.2)	.05
Death	1 (0.1)	3 (0.2)	−0.1	0.3	−6.1 to 5.4	0.5 (0.1 to 4.5)	.51
ED or hospitalization	47 (4.8)	71 (5.8)	−1.0	−0.9	−2.8 to 0.9	0.9 (0.6 to 1.2)	.38
Patients receiving infusion at the same site							
Hospitalization or death	17 (2.4)	4 (0.9)	1.5	1.3	−2.6 to 5.2	2.7 (0.9 to 7.7)	.08
Hospitalization	17 (2.4)	4 (0.9)	1.5	1.3	−2.6 to 5.2	2.7 (0.9 to 7.7)	.08
Death	1 (0.1)	1 (0.2)	−0.1	−0.2	−9.0 to 8.5	0.8 (0.0 to 12.9)	.85
ED or hospitalization	38 (5.3)	26 (5.9)	−0.6	−1.2	−3.8 to 1.4	0.9 (0.6 to 1.5)	.66

Abbreviation: ED, emergency department.

^a^ For patients treated at all health system facilities, patients were treated subcutaneously from July 20 to September 20, 2021, and patients were treated intravenously from July 15 to September 29, 2021. For patients treated at the same health system facilities, patients were treated subcutaneously from September 9 to 29, 2021, and patients were treated intravenously from July 15 to September 29, 2021.

^b^ Model adjusted for age, sex, Black race, and vaccination status.

Among patients treated at clinical sites in which both routes of administration were used, the 28-day risk difference (subcutaneous − intravenous) of hospitalization or death was 1.3% (95% CI, −2.6% to 5.2%; *P* = .50) (Table 3). The corresponding adjusted risk ratio was 2.7 (95% CI, 0.9-7.7; *P* = .08). Adjusted risk differences and risk ratios for death and ED admission or hospitalization were lower in the direction favoring patients treated subcutaneously, but these differences were not significant.

In post hoc supplemental analysis to investigate the statistically higher 28-day risk difference of hospitalization in patients treated subcutaneously, the 28-day risk differences of ICU admission and mechanical ventilation (ie, indicators of severity of hospitalization) were similar in patients treated subcutaneously or intravenously (eTable 5 in the Supplement). In addition, length of stay was similar by route of administration among hospitalized patients.

## Discussion

Among a matched analysis of 1959 patients, subcutaneously administered casirivimab and imdevimab was associated with an estimated 52% lower risk of 28-day hospitalization or death compared with no mAb treatment among EUA-eligible outpatients. Among 2185 patients with mild to moderate COVID-19 treated at an outpatient infusion center, the adjusted risk difference of 28-day hospitalization or death comparing subcutaneous and intravenous mAb treatment was 1.5%, below our predefined similarity boundary of 3%. However, the upper limit of the 95% CI (3.5%) exceeded the clinical boundary, indicating a possible increased risk of hospitalization with subcutaneous mAb administration. However, and although not formally powered for analysis by study design, there was little to no evidence that subcutaneous administration was associated with a higher risk of death or severe hospitalization (ie, ICU admission or mechanical ventilation). Collectively, these data suggest that subcutaneous administration of mAb may be a reasonable alternative to intravenous administration for prevention of death, ICU admission, and need for mechanical ventilation.

To our knowledge, this report is the largest analysis of outpatients with mild to moderate COVID-19 treated with subcutaneously administered mAbs compared with nontreated and intravenously administered mAbs. These noncausal data indicate a consistent, significant benefit of mAb therapy in decreasing hospitalizations and deaths for patients with mild to moderate COVID-19, regardless of route of administration, in a 100% Delta variant landscape. The adjusted risk difference between subcutaneous and intravenous administration for rate of 28-day hospitalization or death was small and not statistically significant, and there was no difference in risk of severity of illness once hospitalized. This evidence is promising because administering intravenous mAb is logistically challenging, and health care systems across the globe continue to face critical staffing shortages amid high SARS-CoV-2–positive patient volumes. Subcutaneous administration of mAb allows for reduced appointment times (because of elimination of need to place a venous catheter and need to infuse the medication for a certain number of minutes), which increases treatment capacity. Indeed, our health care system was able to increase the number of patient appointments for mAb treatment from 400 to 1000 patients per week with the same number of staff by changing the route of administration from intravenous to subcutaneous. In addition, under the Public Readiness and Emergency Preparedness Act in the US, pharmacists are allowed to administer subcutaneous injections, expanding the available staffing pool to much greater capacity.^17^ These important gains in practical resources for stressed health systems must be weighed against the absolute risk difference in hospitalizations with subcutaneous administration and intravenous administration, particularly when assessed in relation to lower risk of hospitalization and death for subcutaneous administration in patients compared with nontreated patients.

Access to safe and effective outpatient treatments for COVID-19 is of critical importance to the global community, and subcutaneous mAb administration has useful implications for scaling resources. By avoiding limitations associated with intravenous administration, subcutaneous mAb treatment and postexposure prophylaxis outpatient treatment location sites can potentially reach disadvantaged neighborhoods and low- and middle-income countries more readily.

### Limitations

Our study has limitations. First, nontreated controls were matched by EUA-eligible risk factors only, and we were unable to determine symptom severity (whether symptomatic or asymptomatic) or vaccination status in these patients. Thus, many nontreated patients may have been asymptomatic and thereby at low risk of hospitalization, which would tend to bias results against mAb treatment. Second, because outcome assessment started on the day of infusion for treated patients yet on the day after a positive SARS-CoV-2 test result for untreated patients, the possibility of immortal time bias exists. However, the sensitivity analysis conducted suggests that if immortal time bias is present, its effect is likely to be small. Third, after September 28, 2021, certain patient populations were prioritized for mAb treatment because of drug and staffing shortages. This prioritization may have resulted in matched nontreated controls having more comorbidities in this period; however, the propensity matching resulted in balanced distribution of both matched and nonmatched covariates. Fourth, although adjusted for statistically, more patients in the subcutaneous group were fully vaccinated compared with the intravenous group at all sites, which may also lower the risk of hospitalization and death. However, the phrase *fully vaccinated* on the referral form meant receipt of 2 doses of an mRNA vaccine or 1 dose of an adenovirus vaccine; further details on time from last dose to mAb referral, type of vaccine, or whether a third primary series dose had been administered to immunocompromised patients were unknown, and therefore *fully vaccinated* cannot be interpreted as fully protected. This difference was also mitigated when the analysis was restricted to patients treated at the same sites. Fifth, the study was conducted before emergence of the Omicron variant; thus, results cannot be generalized to this newer period. Sixth, the mean time from symptom onset to mAb treatment in our study was 6 days. Although these therapies work best earlier in the disease course, administering treatment faster in real-world settings is logistically challenging, and the observed time to treatment in this study represents best practices for mAb treatment across an extensive geographic region. Time-to-treatment windows will be important to consider as novel, oral antiviral medications become available with reduced treatment windows compared with mAb treatment.^18,19^

## Conclusions

In this cohort study of outpatients with mild to moderate COVID-19 symptoms, subcutaneously administered casirivimab and imdevimab was associated with a lower risk of hospitalization or death compared with no mAb treatment. Moreover, no difference was found in the 28-day risk of death, ICU admission, or mechanical ventilation between subcutaneously or intravenously treated patients. Collectively, these results provide preliminary evidence of potential expanded use of subcutaneous mAb treatment, particularly in areas facing treatment capacity and/or staffing shortages.

## Notes

Supplement. **eTable 1.** Monoclonal Antibody Subcutaneous Orders and Reaction Management **eTable 2.** ICD-9 and ICD-10 Codes Used for Diagnoses and Procedures **eTable 3.** Primary and Secondary Outcomes in an Unmatched Cohort of Patients Receiving Subcutaneous Monoclonal Antibody Treatment and an At-Risk Population of Patients Not Receiving Monoclonal Antibody Treatment **eTable 4.** Comparison of Characteristics in Subcutaneous mAb Treated Group and Nontreated Control Group **eTable 5.** 28-Day Hospitalization Outcomes by Route of mAb Administration **eFigure 1.** Distribution of Propensity Scores (X 100) Before and After Matching of Treated and Not Treated Patients **eFigure 2.** Plot of Cumulative Event Rates of Hospitalization/Death by Day of Follow-up (X-Axis) for Matched Treated (Solid Line) and Not Treated (Dashed Line) Patients

## References

[1] O’Brien MP, Forleo-Neto E, Musser BJ, ; Covid-19 Phase 3 Prevention Trial Team. Subcutaneous REGEN-COV antibody combination to prevent Covid-19. N Engl J Med. 2021;385(13):1184-1195. doi:10.1056/NEJMoa2109682 34347950 PMC8362593

[2] Weinreich DM, Sivapalasingam S, Norton T, . REGEN-COV antibody cocktail clinical outcomes study in Covid-19 outpatients. medRxiv. 2021:2021.05.19.21257469. doi:10.1101/2021.05.19.21257469

[3] US Food and Drug Administration. Fact sheet for health care providers emergency use authorization (EUA) of REGEN-COVTM. November 21, 2020. Accessed November 23, 2021. https://www.regeneron.com/downloads/treatment-covid19-eua-fact-sheet-for-hcp.pdf

[4] GOV.UK. Patient information leaflet for Ronapreve. November 19, 2021. Accessed November 23, 2021. https://www.gov.uk/government/publications/regulatory-approval-of-ronapreve/patient-information-leaflet-for-ronapreve

[5] Huang DT, McCreary EK, Bariola JR, . The UPMC OPTIMISE-C19 (optimizing treatment and impact of monoclonal antibodies through evaluation for COVID-19) trial: a structured summary of a study protocol for an open-label, pragmatic, comparative effectiveness platform trial with response-adaptive randomization. Trials. 2021;22(1):363. doi:10.1186/s13063-021-05316-3 34034784 PMC8144687

[6] Global Initiative on Sharing All Influenza Data. Tracking of variants. July 20, 2021. Accessed November 23, 2021. https://www.gisaid.org/

[7] Centers for Disease Control and Prevention. Variants and genomic surveillance for SARS-CoV-2. April 2, 2021. Accessed November 23, 2021. https://www.cdc.gov/coronavirus/2019-ncov/variants/index.html

[8] Bariola JR, McCreary EK, Wadas RJ, . Impact of bamlanivimab monoclonal antibody treatment on hospitalization and mortality among nonhospitalized adults with severe acute respiratory syndrome coronavirus 2 infection. Open Forum Infect Dis. 2021;8(7):ofab254. doi:10.1093/ofid/ofab254 34250192 PMC8241472

[9] Reitz KM, Seymour CW, Vates J, . Strategies to Promote ResiliencY (SPRY): a randomised embedded multifactorial adaptative platform (REMAP) clinical trial protocol to study interventions to improve recovery after surgery in high-risk patients. BMJ Open. 2020;10(9):e037690. doi:10.1136/bmjopen-2020-037690 32994242 PMC7526307

[10] World Health Organization. International Classification of Diseases, Ninth Revision (ICD-9). Geneva, Switzerland: World Health Organization; 1977.

[11] World Health Organization. International Statistical Classification of Diseases, Tenth Revision (ICD-10). Geneva, Switzerland: World Health Organization; 1992.

[12] US Department of Commerce. National Technical Information Service. 2021. Accessed November 23, 2021. https://www.ntis.gov/

[13] Social Security Administration. Social Security master file of Social Security Number holders and applications: death information. October 13, 2021. Accessed November 23, 2021. https://www.ssa.gov/dataexchange/request_dmf.html

[14] Austin PC. An introduction to propensity score methods for reducing the effects of confounding in observational studies. Multivariate Behav Res. 2011;46(3):399-424. doi:10.1080/00273171.2011.568786 21818162 PMC3144483

[15] Rosenbaum PR, Rubin DB. The central role of the propensity score in observational studies for causal effects. Biometrika. 1983;70:41-55. doi:10.1093/biomet/70.1.41

[16] Benchimol EISL, Smeeth L, Guttmann A, ; RECORD Working Committee. The reporting of studies conducted using observational routinely-collected health data (RECORD) statement. PLoS Med. 2015;12(10):e1001885. doi:10.1371/journal.pmed.1001885 26440803 PMC4595218

[17] Department of Health and Human Services. Expanding access to COVID 19 therapeutics—Public Readiness and Emergency Preparedness (PREP) Act declaration. 2021. Accessed November 23, 2021. https://www.phe.gov/Preparedness/legal/prepact/Pages/PREPact-NinethAmendment.aspx

[18] Efficacy and safety of molnupiravir (MK-4482) in non-hospitalized adult participants with COVID-19 (MK-4482-002). ClinicalTrials.gov identifier: NCT04575597. Updated November 4, 2021. Accessed November 23, 2021. https://clinicaltrials.gov/ct2/show/NCT04575597

[19] Evaluation of protease inhibition for COVID-19 in standard-risk patients (EPIC-SR). ClinicalTrials.gov identifier: NCT05011513. Posted August 18, 2021. Accessed November 23, 2021. https://clinicaltrials.gov/ct2/show/NCT05011513

