# Supplementary material for: Association of Subcutaneous or Intravenous Administration of Casirivimab and Imdevimab Monoclonal Antibodies With Clinical Outcomes in Adults With COVID-19
Source: JAMA Netw Open. 2022 Apr 12;5(4):e226920. doi: 10.1001/jamanetworkopen.2022.6920 (PMC9006104; doi:10.1001/jamanetworkopen.2022.6920)
Supplement: Supplement. — eTable 1. Monoclonal Antibody Subcutaneous Orders and Reaction Management eTable 2. ICD-9 and ICD-10 Codes Used for Diagnoses and Procedures eTable 3. Primary and Secondary Outcomes in an Unmatched Cohort of Patients Receiving Subcutaneous Monoclonal Antibody Treatment and an At-Risk Population of Patients Not Receiving Monoclonal Antibody Treatment eTable 4. Comparison of Characteristics in Subcutaneous mAb Treated Group and Nontreated Control Group eTable 5. 28-Day Hospitalization Outcomes by Route of mAb Administration eFigure 1. Distribution of Propensity Scores (X 100) Before and After Matching of Treated and Not Treated Patients eFigure 2. Plot of Cumulative Event Rates of Hospitalization/Death by Day of Follow-up (X-Axis) for Matched Treated (Solid Line) and Not Treated (Dashed Line) Patients [file jamanetwopen-e226920-s001.pdf]

Supplementary Online Content

McCreary EK, Bariola JR, Wadas RJ, et al. Association of subcutaneous or intravenous administration of casirivimab and imdevimab monoclonal antibodies with clinical outcomes in adults with COVID-19. *JAMA Netw Open*. 2022;5(4):e226920. doi:10.1001/jamanetworkopen.2022.6920

eTable 1. Monoclonal Antibody Subcutaneous Orders and Reaction Management

eTable 2. ICD-9 and ICD-10 Codes Used for Diagnoses and Procedures

eTable 3. Primary and Secondary Outcomes in an Unmatched Cohort of Patients Receiving Subcutaneous Monoclonal Antibody Treatment and an At-Risk Population of Patients Not Receiving Monoclonal Antibody Treatment

eTable 4. Comparison of Characteristics in Subcutaneous mAb Treated Group and Nontreated Control Group

eTable 5. 28-Day Hospitalization Outcomes by Route of mAb Administration

eFigure 1. Distribution of Propensity Scores (X 100) Before and After Matching of Treated and Not Treated Patients

eFigure 2. Plot of Cumulative Event Rates of Hospitalization/Death by Day of Follow-up (X-Axis) for Matched Treated (Solid Line) and Not Treated (Dashed Line) Patients

This supplementary material has been provided by the authors to give readers additional information about their work.

eTable 1. Monoclonal Antibody Subcutaneous Orders and Reaction Management

Injection Orders	Reaction Management	Extravasation Management
Inject four subcutaneous injections.	For all reactions:<br>- Obtain vital signs and O2 saturation every 10 minutes.<br>- Refer to orders below for symptomatic management.<br>- Complete RiskMaster Event Report.	Do not administer remainder of injections.<br>- Minor extravasation of non-vesicant/non-irritant solutions do not require additional treatment.<br>- Cold compress may be applied if desired.
Monoclonal antibody orders: casirivimab 600mg – imdevimab 600mg, subcutaneously once (administered as four, 300mg injections)	For fever, chills, pain, or headache:<br>- acetaminophen 1000mg PO once (18 years or older); 650mg PO once (12-17 years)	Closely monitor the patient for 1 hour. Do not administer remaining injections.<br>- The patient's physician (or Advanced Practice Provider, or IV therapy nurse) should be contacted immediately to evaluate the patient if any of the following severe extravasation symptoms occur: i. The patient is experiencing pain ii. The skin around the area is discolored iii. The area is inflamed and/or larger than size of a quarter.
Check vitals at baseline and 30 minutes after injection.	For itching, rash, hives, or flushing:<br>- diphenhydramine 25 mg IV or PO once<br>- famotidine 20 mg IV or PO once<br>- Continuing injections is not recommended	Complete RiskMaster Event, report to FDA Med Watch.
Observe patient for 30 minutes post-injection. Check vitals at end of observation period, prior to discharge.	For MILD shortness of breath, or MILD chest tightness without wheezing or other symptoms CONTACT PHYSICIAN:<br>- Do not administer remainder of injections<br>- Provide supplemental oxygen via nasal cannula to keep O2 saturation >94%<br>- Albuterol metered dose inhaler 2 puffs INH once<br>- Methylprednisolone 60mg IM once (18 years or older); 2mg/kg IVP once (12-17 years, max dose 125mg)	Discharge the patient at the end of the 1-hour monitoring period.
Provide post-administration instructions if adverse reaction or worsening symptoms occur after discharge	For moderate-to-severe respiratory symptoms, stridor, severe bronchospasm, sensation of throat closure or choking, tongue/lip swelling, new onset wheezing, SBP:<br>- Do not administer remainder of injections o Epinephrine 0.3 mg IM (of 1mg/mL solution) once into anterolateral thigh<br>- Place patient into recumbent position with lower extremities elevated<br>- If able to start IV, administer Lactated Ringers 500 mL IV bolus once **FLUSH LINE WITH NS FLUSHES ONLY**<br>- Call "Condition" / Call 911	

© 2022 McCreary EK et al. *JAMA Network Open.*

eTable 2. ICD-9 and ICD-10 Codes Used for Diagnoses and Procedures

Diabetes						
250.91	250.93	E11.59	E13.3399	E11.3392	E10.49	E13.3523
250.23	250.22	E11.8	E11.3299	E10.36	E11.621	E10.649
250.10	250.40	E10.37X9	E11.3312	E10.8	E13.00	E13.37X2
E13.620	250.63	E13.43	E10.3492	E13.3212	E10.9	E13.3551
E13.9	250.71	E13.3213	E10.3499	E13.3522	E10.65	E11.3591
E13.3599	250.70	E13.3593	250.72	E11.641	E11.3511	E10.3512
E11.3291	E13.3393	E10.3212	250.83	E10.21	E10.622	E13.69
E11.3541	E11.39	E10.3549	E10.43	E11.51	E11.00	E10.37X1
E10.3493	E10.3543	E13.311	E13.11	E13.01	E11.3413	E13.3531
E10.51	E10.3591	E13.3313	E10.29	E11.11	E10.3299	E13.3592
E13.3292	E11.42	E11.3543	E10.3312	E11.3533	E10.3411	E13.49
E13.3419	E10.628	E10.3393	E13.3542	E11.3553	E13.3493	E11.3219
E13.59	E11.10	E10.3519	E10.39	E10.311	E13.3532	E10.3313
E10.630	E11.3491	250.90	E11.628	E10.3513	E13.3549	E11.3419
E11.37X2	E10.3399	250.20	Z96.41	E10.41	E13.3559	E11.630
E10.3521	E13.36	250.80	E13.3312	E11.43	E11.3393	E13.3491
E13.3552	E11.65	250.50	E13.3392	E13.638	E10.3599	E13.3519
E13.40	E10.3532	250.61	E11.22	E10.3531	E11.649	E13.3539
E13.52	E13.41	250.62	E11.29	E10.3592	E11.3559	E11.37X3
E13.622	E11.3213	E13.630	E11.3212	E13.3512	E10.3391	E10.3523
E11.3311	E11.3313	E13.3219	E11.3552	E13.42	E10.52	E10.3529
E11.3512	E13.641	E10.10	E11.49	E13.44	E11.620	E10.42
E10.3511	E10.37X2	E11.21	E13.21	E11.3399	E10.3293	E13.65
E13.3411	E11.37X9	E11.3211	E11.01	E11.3492	250.41	E13.610
E11.3513	E10.3319	E11.3593	E10.641	E11.3532	250.12	E11.3493
E10.3219	E11.69	E10.319	E13.621	E11.3549	250.21	E11.622
E11.41	E11.3293	E13.3492	E11.3319	E10.3292	250.02	E13.3391
E13.3319	E13.3293	E13.649	E11.3519	E10.3553	250.13	E10.638
E10.11	E11.3542	E11.9	E11.3522	E13.22	E13.3299	E13.3541
E10.3551	E13.3291	E10.3593	E10.3491	E13.29	E13.3543	E11.3521
E11.618	E13.3591	E10.621	E10.618	E11.3551	E13.37X9	E11.3523
E11.3529	E10.3291	E13.3533	E13.3529	E13.3413	E11.3412	E10.3522
E13.3211	E10.3392	E13.628	E13.8	E13.3521	E10.3419	E13.3511
E13.3311	E11.319	E10.22	E10.44	E13.618	E10.40	E13.37X1
E13.3553	E11.3391	E11.36	E13.37X3	E11.3411	250.60	E10.59
E11.311	E10.3559	E10.3211	E10.3541	E10.610	250.92	E13.10
E11.37X1	E13.51	E10.3539	E10.620	E11.52	250.81	V45.85
E11.44	E11.3499	E10.37X3	250.03	E11.3292	E13.39	E11.3531
250.32	E10.3412	E11.638	250.00	E11.40	E11.3599	E10.69
250.52	E10.3533	250.73	250.82	E10.3552	E10.3213	E13.3412
250.53	E11.610	250.11	250.01	E11.3592	E13.319	E11.3539

© 2022 McCreary EK et al. *JAMA Network Open.*

250.42	250.33	250.43	250.30	E10.3311	250.51	E10.3413
E13.3499	E10.3542	250.31	250.31	..	..	..
Obstructive Sleep Apnea						
327.29	G47.33	G47.31	327.20	786.03	G47.39	327.23
G47.37	G47.30	R06.81	327.27	327.21	780.51	780.53
780.57	..	..	..	..	..	..
Dyspnea						
786.09	R06.02	R06.00	786.05	R06.09	..	..
Asthma						
493.10	Z91.849	O9A.212	J44.1	O9A.113	Z57.1	Z91.843
493.11	Z57.7	J20.1	T50.995A	Z57.5	Z57.2	J45.22
648.93	466.0	J45.32	O99.283	O9A.312	493.21	799.02
J44.9	493.12	O9A.211	O9A.313	O9A.411	491.9	465.9
J45.902	493.20	Z77.22	O99.619	493.01	J45.40	493.82
O99.519	648.90	O99.820	Z56.4	493.92	Z56.5	493.91
O99.281	O9A.319	J45.990	O25.11	R09.02	Z56.6	J45.31
O99.712	J45.41	O25.10	J42	Z91.82	J20.8	O99.612
O9A.112	O9A.412	J06.9	O25.12	J20.4	Z77.122	J45.30
O99.713	O99.613	J20.2	Z91.841	O9A.413	J45.909	O99.611
Z91.89	Z56.9	O25.13	493.22	O9A.512	Z57.6	Z77.21
J45.991	O99.512	J45.52	995.27	O9A.213	J20.5	Z57.0
Z56.81	Z77.128	493.00	V15.89	Z57.9	O9A.219	J20.9
J45.21	J20.7	V62.1	Z57.39	O9A.511	Z77.112	O99.511
O99.280	Z91.842	493.90	O99.719	Z92.89	Z77.9	Z77.111
O99.513	Z56.3	J45.20	Z92.84	J20.3	O99.282	O9A.519
Z77.118	J20.0	O9A.119	J45.50	Z57.31	O9A.111	J33.9
J45.51	471.9	Z77.123	J45.998	J45.42	Z56.2	Z57.8
J45.901	493.81	Z56.89	Z77.110	O9A.419	Z57.4	120.6
O9A.513	493.02	O99.711	J44.0	O9A.311	..	..
Pulmonary Hypertension						
I27.82	I27.0	I27.20	416.0	I27.23	I27.22	I27.89
I27.29	416.2	I27.24	416.8	I27.21	..	..
Hypertension						
401.9	593.82	I15.1	405.09	I15.9	405.99	593.89
401.1	405.19	I15.2	401.0	N28.89	405.11	I15.0
I16.0	405.01	I16.1	I15.8	405.91	I10	I16.9
Chronic Obstructive Pulmonary Disease						

496	J47.0	492.8	494.0	J41.8	J45.42	491.20
493.10	493.12	491.8	J45.20	J43.1	493.21	J45.31
493.11	493.20	J45.32	J44.1	J41.1	491.9	J45.30
J44.9	494.1	J43.9	J43.8	J45.50	J45.40	J43.2
J43.0	491.21	J45.52	J42	J44.0	J47.1	491.1
J45.21	J45.41	491.22	493.22	493.01	J45.22	J47.9
J45.51	493.02	493.00	492.0	..	..	..
Atrial Fibrillation						
427.32	I48.0	I48.4	I48.92	I48.1	427.31	I48.3
I48.2	I48.91	..	..	..	..	..
AGC Valvular Heart Disease						
115.04	I39	I34.9	I37.0	I35.0	424.1	424.3
I36.8	I37.1	I36.9	I34.0	I34.8	424.91	424.2
I35.9	I38	424.99	I35.1	I35.2	115.14	I34.2
I37.8	I35.8	421.1	I36.0	I36.2	424.0	I34.1
I37.2	424.90	I36.1	115.94	I37.9	..	..
Coronary Artery Disease						
414.02	I25.119	I25.711	I25.750	I25.42	I25.798	I25.738
414.10	I25.82	I25.769	I25.812	I25.89	I25.730	I25.710
I25.718	I25.811	I25.10	I25.110	I20.8	I25.3	414.06
I25.791	I25.83	413.9	414.01	I25.731	I25.761	414.07
I25.111	I25.701	414.9	414.12	I20.1	I25.9	414.04
I25.708	414.00	413.1	414.2	413.0	I25.721	I25.84
I25.729	414.3	I20.9	414.19	414.03	I25.700	I25.790
I25.739	I25.760	I25.751	414.11	414.4	I25.118	I25.709
414.8	I25.799	I25.5	414.05	I25.41	I25.768	I25.759
I25.758	I25.810	I25.728	I25.719	I25.6	I25.720	..
Stroke						
433.00	I60.31	803.26	433.01	801.21	I60.4	800.79
433.91	I62.1	I63.532	434.11	804.26	I60.8	997.02
800.26	G45.9	I60.52	801.76	800.29	I61.5	852.36
852.19	I63.113	I63.12	453.86	803.71	I63.331	803.21
286.7	434.91	I65.21	I65.22	342.82	I63.6	434.90
803.29	800.21	H34.11	I66.9	803.76	I67.6	852.11
432.1	801.26	I63.411	I60.2	800.71	I60.9	432.9
852.09	433.11	I63.233	I63.131	433.10	I63.012	437.6
I60.01	800.20	I63.031	I66.01	800.70	I63.421	I61.4
I63.511	342.81	I63.10	I62.00	I63.02	I63.22	I63.20
D68.32	342.92	I77.0	S06.6X0A	I63.112	I66.02	I63.423

© 2022 McCreary EK et al. *JAMA Network Open.*

G81.94	435.3	I60.00	I82.C12	I63.59	435.0	I63.541
I60.11	852.01	Q28.2	I63.533	I63.132	435.1	I60.32
I62.02	852.26	804.20	I65.02	I63.211	433.21	I63.8
I65.03	801.20	801.71	852.10	I63.213	804.70	I69.354
I63.312	447.0	852.00	853.01	I60.50	804.71	G81.91
I63.413	803.20	I60.30	453.40	I69.898	804.79	I63.232
I63.513	H34.232	I65.1	336.1	I60.7	433.31	I63.522
I63.543	I63.531	I63.432	I61.2	I65.23	432.0	I63.39
I61.9	I62.03	I63.539	I61.3	I60.02	801.29	I63.441
I63.032	I62.9	453.82	I61.6	I60.12	803.70	I63.442
I62.01	I63.419	342.91	I63.231	I61.8	804.29	I60.10
I63.412	I63.311	362.32	I63.29	I63.111	801.79	852.39
I63.521	I63.422	433.81	I63.50	I63.542	I61.0	804.21
I97.820	I63.011	434.00	I60.6	S06.5X9A	I63.322	I63.212
I63.40	I69.298	435.9	I61.1	I63.9	I63.512	I82.622
804.76	I63.321	801.70	I65.01	852.29	G95.11	438.89
747.81	I63.49	803.79	I63.431	800.76	I63.449	430
433.20	H34.231	362.31	G45.0	431	I82.401	S06.340A
434.01	I63.30	438.22	434.10	I60.51	..	..
Congestive Heart Failure						
417.9	425.7	I27.29	428.20	I50.84	I28.0	I50.83
425.8	428.43	I50.812	398.91	417.8	I42.3	I11.0
425.9	I42.0	I27.1	429.0	428.30	I42.1	I50.33
425.5	I50.811	I50.814	I28.8	428.33	I50.21	I27.23
416.9	I43	I50.9	I27.20	404.93	I42.4	A18.84
428.42	I26.01	I27.0	I50.41	416.0	425.0	I09.81
I13.2	I50.89	I27.81	I42.8	402.91	428.21	415.0
I50.30	I26.02	I13.0	404.11	416.1	428.22	428.41
I51.5	I50.31	I42.2	I42.7	425.4	416.8	402.11
I50.20	I51.4	404.13	I50.813	404.01	425.3	417.1
I50.42	428.31	429.1	I42.5	I50.32	425.2	428.40
I42.9	402.01	417.0	I50.23	I50.82	428.9	I27.21
428.1	404.91	428.0	I50.810	I50.43	428.23	I27.22
428.32	I28.9	404.03	I50.40	126.09	142.6	I50.22
425.18	I27.9	425.11	I27.24	128.1	150.1	..
Chronic Kidney Disease Stage 3						
585.3	N18.32	N18.3	N18.30	N18.31	..	..
Chronic Kidney Disease Stage 4						
N18.4	585.4	..	..	..	..	..

© 2022 McCreary EK et al. *JAMA Network Open.*

Chronic Kidney Disease Stage 5						
N18.5	585.5	..	..	..	..	..
End Stage Renal Disease						
585.6	N18.6	V45.12	V45.11	Z99.2	Z91.15	..
Cancer						
148.2	205.81	C44.92	C93.Z0	140.4	C01	C91.Z0
173.01	208.82	C72.22	C4A.4	144.1	C15.8	C7B.04
173.62	209.23	C83.18	C4A.52	150.5	C48.0	C94.40
173.71	209.25	C83.99	C08.1	162.2	C34.80	C15.3
182.8	142.0	C82.91	C10.4	162.8	C44.611	C18.9
183.5	147.2	C82.43	C25.0	170.5	C44.711	C31.3
188.1	151.5	C91.40	C44.591	173.09	C44.80	C34.10
196.3	153.7	C84.A3	C83.34	173.90	C44.81	C34.31
197.5	173.52	C85.13	C83.85	187.6	C62.91	C38.3
238.79	201.47	C93.11	C81.08	198.81	C66.1	C47.6
160.2	201.75	C7A.8	C82.63	202.36	C69.32	C43.20
173.12	202.98	C02.2	C82.37	202.74	C70.0	C44.01
173.29	203.12	C03.9	C85.80	204.82	C73	C50.412
200.54	207.82	C16.1	C95.00	208.11	C83.32	C50.022
201.53	209.22	C31.0	C95.12	209.14	C83.53	C46.7
204.22	153.5	C83.52	C4A.10	170.4	C83.91	C71.5
205.10	157.2	C83.81	C02.1	176.2	C83.05	C76.51
209.13	172.4	C83.88	C07	196.5	C81.09	C76.8
209.73	173.59	C83.16	C13.1	198.82	C82.40	C83.87
153.1	173.60	C83.96	C24.9	200.65	C82.32	C81.70
170.1	197.8	C81.16	C25.3	203.01	C82.13	C81.48
173.91	200.24	C84.42	C48.1	204.81	C82.35	C81.28
196.6	201.77	C92.00	C47.21	205.00	C82.96	C81.96
200.38	201.90	C7A.093	C44.219	172.8	C84.41	C82.29
201.12	205.92	C02.3	C44.320	181	C4A.21	C84.A8
201.22	206.20	C39.9	C46.52	187.3	C06.9	C91.51
201.52	207.00	C44.319	C53.8	200.86	C34.12	C92.A0
202.91	209.11	C57.21	C57.02	202.96	C43.30	C92.90
173.19	160.8	C69.22	C83.83	206.92	C44.112	C94.42
180.0	184.1	C75.2	C83.11	142.8	C44.212	C05.2
185	190.9	C80.0	C83.06	143.9	C44.509	C22.9
202.37	200.05	C83.19	C81.97	176.5	C44.629	C40.92
204.00	200.31	C84.67	C82.99	200.30	C50.611	C47.20
209.20	201.76	C82.82	C82.03	201.70	C50.819	C44.91
209.26	202.63	C82.23	155.2	201.94	C50.329	C50.112
209.27	204.12	C85.14	161.0	202.54	C46.50	C58

© 2022 McCreary EK et al. *JAMA Network Open.*

209.32	205.30	C85.96	170.3	202.84	C60.1	C51.0
147.8	C00.3	C88.2	196.1	C00.8	C68.0	C63.00
148.0	C03.0	C91.A2	201.15	C44.192	C79.32	C69.01
150.8	C10.2	C7A.026	146.9	C44.89	C81.46	C79.10
172.2	C10.3	C4A.31	148.9	C46.2	C81.20	C83.00
183.3	C13.0	C04.0	162.5	C69.60	C81.29	C81.34
189.8	C13.2	C34.92	173.22	C69.11	C84.00	C82.93
200.56	C16.2	C40.01	173.31	C79.62	C84.11	C85.90
201.10	C16.6	C44.590	189.0	C83.50	C84.18	C91.01
201.43	C21.0	C44.602	196.2	C83.51	C85.10	C91.A0
201.66	C38.4	C61	197.4	C85.20	C84.98	C94.02
202.12	C43.8	C67.9	200.42	C81.13	C96.4	C95.01
202.32	C50.612	C74.02	202.24	C81.24	C91.60	C95.90
202.82	C50.429	C78.80	202.85	C82.86	C15.5	C4A.22
202.83	C53.9	C83.30	204.10	C82.56	C48.2	C4A.62
203.00	C77.9	C83.97	207.01	C88.3	C44.00	C23
207.20	C79.72	C81.36	209.31	C93.30	C44.500	C49.22
150.0	C81.03	C82.00	209.72	C95.11	C50.519	C44.99
184.8	C82.41	C82.81	146.1	C7B.02	C50.321	C50.221
190.7	C86.2	C84.Z7	151.0	C09.9	C67.6	C65.1
200.50	C85.87	C85.88	174.9	C14.8	C83.04	C77.3
200.61	C82.58	C7A.090	183.0	C34.81	C81.94	C81.72
201.18	C92.50	C49.3	190.5	C39.0	C82.17	C82.80
202.60	C93.91	C46.9	194.5	C40.91	C82.87	C82.02
209.12	C94.81	C56.9	200.74	C44.119	C82.28	C4A.51
159.8	C7A.022	C69.61	200.83	C72.30	C91.61	C16.0
165.0	C12	C69.50	201.27	C72.40	C92.61	C40.81
191.4	C14.2	C78.5	202.03	C72.1	C88.0	C43.12
191.8	C34.02	C82.51	202.31	C70.1	C05.9	C43.61
200.46	C40.21	C91.30	202.41	C77.1	C06.89	C43.9
200.51	C43.72	C92.32	202.62	C79.01	C11.8	C44.511
200.64	C44.729	C00.4	204.20	C83.73	C41.2	C50.411
202.34	C44.90	C06.80	207.21	C84.69	C44.191	C50.229
207.02	C50.822	C34.82	145.5	C83.94	C44.692	C53.0
141.3	C54.0	C44.619	154.8	C81.76	C44.82	C57.10
144.0	C71.0	C50.211	160.0	C81.38	C50.912	C63.02
144.8	C72.20	C50.311	171.9	C82.07	C63.9	C72.21
148.8	C72.32	C54.1	173.82	C84.Z0	C67.1	C74.00
173.99	C79.19	C69.21	197.2	C85.82	C67.3	C75.3
174.6	C81.31	C69.81	200.36	C82.53	C76.40	C75.8
200.08	C82.69	C72.31	141.0	C92.A2	C83.38	C77.2
200.57	C84.09	C74.01	141.2	C4A.8	C84.63	C79.11
201.16	C84.Z5	C79.31	151.4	C96.5	C85.28	C83.72

201.50	C91.00	C83.12	157.9	C10.1	C81.10	C83.80
202.17	C92.40	C84.78	171.8	C14.0	C82.30	C84.68
202.22	C7A.010	C81.91	173.50	C31.9	C84.16	C82.84
203.11	C7B.03	C84.05	187.2	C37	C84.46	C82.47
207.80	C00.6	C4A.71	190.1	C40.00	C86.0	C85.18
209.35	C32.2	C00.0	192.8	C47.10	C84.Z2	C96.Z
C09.1	C40.12	C18.4	196.8	C49.A3	C82.57	C90.20
C26.9	C49.A2	C22.2	198.3	C44.390	C92.41	C90.31
C32.9	C47.9	C34.01	201.00	C44.621	C7A.019	C92.31
C34.32	C44.109	C67.8	146.7	C60.2	C4A.70	C84.12
C38.1	C44.229	C64.2	157.1	C69.40	C11.2	C84.A1
C57.11	C44.519	C69.92	157.4	C69.62	C49.0	C84.Z4
C79.61	C50.911	C74.11	170.9	C72.59	C44.309	C82.55
C84.70	C79.89	C75.4	176.4	C83.35	C50.121	C90.32
C84.79	C83.03	C75.5	183.4	C84.74	C57.12	C86.6
C82.36	C92.20	C88.4	186.0	C81.39	C51.8	C81.21
C84.19	C94.00	C81.06	201.02	C96.21	C69.12	C85.19
C84.17	C95.10	C81.47	201.63	C84.A9	C77.5	C91.Z2
C96.20	C96.6	C81.27	202.27	C91.90	C83.14	C7A.012
C94.22	C17.0	C82.45	204.01	C21.1	C81.78	202.71
C4A.72	C47.0	C82.27	204.21	C21.8	C81.25	208.00
C7B.09	C43.60	C85.17	209.21	C48.8	C81.92	208.81
C40.82	C44.300	C90.10	149.8	C49.11	C84.99	208.92
C44.520	C50.511	C90.21	173.10	C44.122	C85.15	209.70
C67.4	C50.929	144.9	173.40	C44.292	C86.1	C81.40
C69.02	C53.1	156.2	190.8	C50.419	C91.31	C82.42
C75.0	C54.9	173.00	202.45	C46.4	C7B.1	C84.92
C83.13	C64.1	179	205.11	C57.4	C7B.8	C84.A5
C84.64	C74.91	192.1	145.8	C51.2	C47.8	C92.42
C82.15	C83.15	195.5	147.1	C74.92	C44.09	C86.3
C84.95	C84.61	197.1	151.6	C78.39	C44.699	C16.9
C90.02	C84.71	198.4	153.8	C83.79	C50.512	C49.20
C91.11	C81.17	201.01	159.1	C83.10	C57.00	C44.222
C93.Z2	C81.35	201.26	160.4	C84.73	C63.10	C44.42
C11.0	C82.19	201.48	161.3	C82.20	C69.30	174.3
C49.4	C82.90	201.67	180.9	C82.60	C75.9	198.0
C43.59	C82.04	202.01	183.2	C82.18	C78.7	200.17
C44.291	C82.85	202.35	200.03	C82.68	C81.32	200.76
C44.702	C84.07	202.67	201.06	C82.59	C82.95	201.13
C50.029	C84.14	161.9	201.20	C88.8	C84.Z1	C7A.095
C50.821	C84.91	187.1	202.50	C92.12	C91.12	C13.8
C50.829	C85.11	200.04	203.10	C94.21	C94.20	C34.91
C54.2	C92.22	200.13	205.02	C15.4	C7A.025	C47.22

C84.65	C94.01	200.37	206.90	C40.11	C22.0	C44.622
C82.61	C4A.11	200.41	C40.10	C50.529	C25.4	C50.811
C91.41	C17.8	201.25	C40.20	C56.1	C50.322	C56.2
C82.50	C34.30	202.11	C43.0	C81.14	C57.20	C63.7
C85.84	C40.80	202.87	C43.70	C82.62	C51.1	C74.90
C85.86	C47.12	141.1	C50.129	C82.24	C69.10	C79.52
C88.9	C49.9	183.8	C63.01	C85.91	C69.82	C81.41
C92.02	C46.51	189.9	C67.2	C7A.029	C78.2	C84.13
C08.9	C79.51	199.0	C69.51	C7A.096	C79.2	C96.29
C17.3	C79.71	200.80	C72.41	C4A.0	C85.27	C84.45
C18.3	C85.26	201.62	C83.76	C4A.61	C81.79	C84.97
C32.8	C90.00	201.91	C85.24	C02.4	C82.92	C90.01
C43.51	C93.31	201.98	C81.02	C08.0	C82.05	C17.2
C43.71	C00.1	202.44	C82.09	C06.1	C84.10	C43.22
C50.621	C25.9	205.80	C82.01	C33	C84.48	C44.311
C83.58	C44.199	162.9	C82.25	C34.00	C85.83	C44.510
C83.84	C50.019	164.8	C84.Z3	C38.2	C92.01	C83.33
C81.43	C55	184.4	C92.60	C41.3	C93.32	C83.93
C81.11	C54.3	190.4	C92.91	C44.521	C7A.00	C83.08
C81.18	C62.11	191.0	C49.21	C44.712	C4A.39	C82.22
C81.90	C77.0	200.52	C47.4	C46.1	165.8	164.2
C82.10	C85.21	205.12	C49.A5	C68.8	170.8	173.21
C94.31	C83.90	151.3	C43.11	C76.2	171.3	173.70
C09.8	C86.5	156.1	C43.31	C79.9	188.8	194.3
C18.1	C83.01	160.3	C44.102	C84.44	200.21	194.8
C49.10	C83.98	161.2	C44.612	C92.Z0	200.28	201.05
C43.21	C81.42	162.0	C50.122	C92.Z2	202.38	202.28
C44.121	C81.95	165.9	C57.8	C94.82	206.02	202.93
C44.209	C84.94	187.8	C62.10	C49.A4	173.92	205.21
C44.701	C91.50	200.02	C62.92	C43.39	194.9	206.91
C50.119	C7A.094	201.92	C63.8	C44.211	198.5	208.01
C50.619	C4A.9	204.02	C78.01	C50.522	200.18	208.90
C65.2	C7B.00	209.15	C79.81	C76.3	200.63	C13.9
C69.52	C03.1	154.0	C83.55	C78.30	200.70	C22.7
C69.00	C16.8	172.5	C83.02	C79.00	200.82	C32.3
C69.91	C22.8	175.0	C82.49	C84.60	201.17	C38.8
C71.6	C25.1	201.24	C82.83	C84.62	203.02	C47.5
C80.2	C41.9	201.65	C96.22	C84.72	152.9	C44.719
C85.22	C44.129	202.18	C90.12	C81.00	184.3	C46.0
C81.71	C50.422	143.1	C49.A9	C81.49	188.7	C52
C81.75	C69.80	147.3	C43.52	C82.14	195.1	C60.9
C81.07	C76.52	150.1	C44.299	C84.43	197.0	C69.20
C96.A	C83.56	171.6	C44.529	C85.85	201.55	C74.12

© 2022 McCreary EK et al. *JAMA Network Open.*

C91.42	C82.67	173.51	C50.521	C85.16	202.46	C84.66
C90.22	C84.04	174.2	C54.8	C7A.024	202.95	189.3
C92.30	C96.0	192.2	C78.6	C7A.092	206.11	196.0
C93.12	C84.49	201.71	C81.73	C22.3	209.02	200.01
C4A.30	C92.52	202.66	C81.05	C25.2	143.8	200.25
C05.0	C7A.011	202.81	C81.30	C34.2	150.4	200.75
C05.1	C00.9	206.10	C82.34	C50.012	151.9	202.13
C22.4	C16.4	208.22	C82.94	C50.319	152.1	207.22
C24.0	C31.2	145.1	C84.90	C50.921	190.3	208.02
C49.A1	C41.1	145.4	C91.52	C67.0	195.8	209.29
C44.41	C50.011	145.9	C4A.60	C81.15	200.45	209.75
C44.721	C50.622	146.2	C04.8	C82.16	200.73	154.3
C51.9	C78.02	153.4	C06.2	C84.08	202.21	160.5
C57.9	C85.23	156.9	C15.9	C85.89	202.64	164.0
C62.02	C85.97	158.8	C16.5	C85.98	209.03	184.9
C71.4	C94.6	171.5	C18.8	C91.Z1	141.6	195.3
C45.7	141.9	172.6	C24.8	C93.90	196.9	202.58
C83.74	162.4	173.39	C26.0	C4A.59	200.32	148.1
C82.65	164.1	191.5	C31.1	140.5	200.35	155.0
C82.97	174.1	198.1	C38.0	156.0	201.58	159.0
C82.38	200.22	200.44	C44.792	163.8	202.06	171.7
C82.98	200.48	200.77	C50.222	173.49	202.42	174.0
C90.30	200.62	201.07	C50.421	174.5	202.47	194.1
C92.Z1	201.11	201.74	C79.49	200.68	204.91	200.53
C93.02	202.15	202.52	C79.60	201.23	145.3	202.14
C94.80	202.57	206.80	C92.A1	203.81	150.3	C02.0
C7A.1	202.86	206.81	C7A.021	209.74	152.0	C22.1
C02.8	206.01	C20	C10.0	142.9	157.3	C44.399
C40.30	273.3	C32.1	C30.1	143.0	160.1	C44.40
C49.A0	140.1	C40.90	C41.4	173.89	172.9	C50.212
C49.6	146.3	C49.12	C44.221	188.9	182.1	C50.922
C44.02	191.2	C49.8	C44.709	191.7	187.9	C57.01
C44.501	194.6	C43.10	C50.812	200.20	190.6	C69.42
C44.609	195.2	C44.791	C63.11	202.43	200.12	C75.1
C44.691	198.6	C46.3	C64.9	202.76	201.78	C78.89
C62.01	200.14	C62.00	C76.41	205.22	201.96	C83.07
C71.8	200.88	C70.9	C78.1	206.82	202.05	C81.77
C76.1	201.14	C83.75	C79.40	209.00	202.08	C81.23
C83.59	201.21	C81.22	C84.76	145.6	202.20	C82.31
C82.39	201.57	C82.21	C83.92	146.5	202.92	C85.12
C82.26	202.94	C82.33	C81.33	149.1	206.12	C93.00
C84.Z6	208.80	C82.08	C82.11	152.8	141.4	C10.9
C84.A7	208.91	C85.99	C84.A0	173.79	154.2	C40.22

© 2022 McCreary EK et al. *JAMA Network Open.*

C91.92	140.0	C82.54	C96.9	180.8	163.1	C44.111
C7A.023	147.0	C84.A4	C09.0	195.4	173.20	C44.301
C94.41	149.9	C95.02	C11.9	200.16	173.30	C44.391
C16.3	164.3	C7A.098	C19	200.27	184.2	C63.12
C18.5	192.0	C7B.01	C25.7	201.73	191.1	C72.42
C34.11	201.28	C11.1	C45.1	202.23	197.6	C76.50
C45.0	202.40	C49.5	C26.1	202.78	197.7	191.6
C45.2	202.65	C43.4	C44.601	208.20	198.2	200.00
C40.32	204.90	C43.62	C44.799	141.8	201.72	200.34
C47.3	206.00	C50.021	C71.1	142.2	202.48	200.85
C44.201	209.10	C62.12	C71.3	153.9	203.82	200.87
C69.31	209.36	C60.0	C72.50	155.1	204.11	201.60
C69.90	209.79	C65.9	C84.75	158.9	205.01	202.02
C78.00	146.8	C74.10	C83.95	170.7	205.82	202.30
C83.54	150.9	C76.0	C82.46	187.4	157.8	202.73
C83.89	153.6	C78.4	C85.81	187.5	161.8	209.17
C83.82	173.32	C79.02	C91.32	188.5	173.42	209.71
C81.74	175.9	C80.1	C05.8	200.40	197.3	C17.9
C81.26	200.71	C82.66	C11.3	200.66	199.2	C18.7
C81.37	201.41	C84.Z9	C47.11	201.04	200.10	C30.0
C82.89	204.80	C86.4	C44.321	201.64	200.72	C44.202
C84.03	172.1	C85.93	C44.49	202.25	200.78	C57.3
C84.A2	176.3	C91.91	C67.7	202.97	200.81	C57.22
C91.02	191.9	C92.51	C69.41	205.91	201.56	C66.9
C92.62	195.0	C06.0	C71.9	140.3	202.75	C71.7
C92.10	200.47	C24.1	C77.4	151.1	C00.5	C83.39
C92.92	200.60	C44.310	C83.71	164.9	C02.9	C82.44
C93.01	200.84	C62.90	C83.78	171.4	C10.8	C82.06
C93.Z1	201.44	C68.1	C84.77	173.80	C18.2	C84.01
140.8	202.04	C72.0	C81.98	180.1	C34.90	C82.52
151.8	202.10	C83.31	C82.12	188.3	C44.101	200.58
163.9	202.33	C83.70	C84.06	192.3	C44.722	201.03
173.72	202.80	C83.77	C91.10	201.42	C50.219	201.54
184.0	205.20	C85.29	C91.A1	209.01	C60.8	201.93
200.11	209.16	C85.25	C7A.091	151.2	C83.17	201.97
201.61	142.1	C81.19	C4A.12	153.0	C83.09	202.90
201.68	149.0	C82.48	C4A.20	171.0	C81.01	205.90
202.26	153.2	C82.88	C04.9	173.11	C81.45	207.81
202.55	170.2	C90.11	C50.312	173.69	C82.64	148.3
203.80	174.4	C94.32	C71.2	188.2	C85.94	153.3
156.8	176.1	C00.2	C79.82	190.0	C84.A6	172.3
159.9	186.9	C04.1	C45.9	199.1	C93.92	176.9
161.1	194.4	C18.6	C83.86	200.06	C95.92	191.3

© 2022 McCreary EK et al. *JAMA Network Open.*

176.8	205.32	C31.8	C81.44	200.55	C17.1	193
183.9	208.12	C32.0	C81.12	204.92	C18.0	194.0
192.9	209.34	C40.02	C81.99	209.24	C21.2	198.89
198.7	140.6	C40.31	C85.95	209.30	C25.8	200.26
200.07	145.0	C44.329	C84.Z8	154.1	C41.0	200.43
201.46	147.9	C50.111	C92.21	173.02	C44.599	202.70
201.95	150.2	C50.919	C95.91	173.61	C63.2	202.72
202.53	170.6	C50.629	140.9	174.8	C67.5	206.21
202.61	171.2	C57.7	146.0	182.0	C66.2	141.5
202.68	172.7	C79.70	200.33	189.4	C68.9	163.0
208.21	176.0	C83.37	201.40	200.15	C72.9	173.81
209.33	188.4	C83.57	201.51	200.67	C76.42	C7A.020
145.2	189.2	C81.04	202.56	201.08	C77.8	152.2
146.6	190.2	C84.02	202.77	202.00	C83.36	173.41
158.0	201.45	C84.15	205.31	202.07	C81.93	C94.30
170.0	202.16	C84.40	152.3	202.51	C84.47	189.1
172.0	208.10	C85.92	157.0	202.88	C84.96	200.23
187.7	146.4	C84.93	160.9	206.22	C92.11	C93.10
188.0	188.6	C91.62	162.3	277.89	..	..
Fatty Liver Disease						
K75.81	K76.89	571.8	K70.0	571.0	K76.0	..
Allergic Rhinitis						
472.0	J30.1	477.0	477.2	J30.0	477.9	477.1
J30.2	J30.9	J31.0	J30.81	477.8	J30.5	J30.89
Rheumatoid Arthritis						
M05.429	M05.679	M06.849	M05.071	M05.272	M05.871	M06.259
M05.459	M05.431	M05.532	M05.09	M06.042	M06.272	M05.029
M05.711	M05.829	M05.59	M05.211	M05.312	M06.871	M06.072
M05.279	M05.832	M05.252	M05.631	M05.362	M06.041	M06.331
M05.661	M06.032	M05.369	M05.652	M05.629	M06.069	M05.159
M05.671	M06.052	M05.372	M06.08	M05.172	M05.331	M05.469
M05.571	M06.062	M06.859	M06.09	M05.511	M05.662	M05.831
M06.212	M05.19	M05.621	M05.259	M05.432	M05.439	M06.839
M06.29	M05.839	M05.529	M05.162	M05.452	M05.742	M05.339
M05.649	M06.371	M06.011	M05.451	M06.321	M06.20	M05.612
M06.221	M05.012	M06.279	M05.749	M06.362	M06.322	M05.179
M06.872	M05.639	M05.361	M05.759	M05.212	M05.322	M05.872
M05.161	M05.519	M05.659	M05.79	M06.079	M05.152	M06.211
M05.741	M05.539	M05.719	M05.061	M05.232	M05.70	M05.30
M06.369	M05.89	M06.251	M06.232	M05.271	M05.849	M05.522

© 2022 McCreary EK et al. *JAMA Network Open.*

M05.521	M06.229	M06.80	M06.28	M05.672	M06.841	M05.472
M05.9	M06.39	M05.29	M06.819	M05.131	M06.861	M06.021
M06.339	M05.121	M05.561	M05.219	M05.512	M05.111	M06.269
M05.311	M05.531	M05.771	M05.39	M05.722	M06.029	M05.169
M05.319	M06.359	M06.061	M05.141	M06.262	714.81	M05.562
M06.4	M06.852	M06.89	M05.732	M05.151	714.2	M06.231
M06.012	M05.541	M05.019	M05.822	357.1	M05.579	M05.049
M06.022	M06.851	M05.072	M06.352	M05.712	M05.769	M05.852
M05.231	M05.032	M05.869	M06.862	M05.721	M05.851	M06.049
M05.241	M05.619	M06.88	M06.869	M05.842	M05.039	M06.249
M05.112	M05.40	M05.341	M05.641	M06.071	M05.332	M06.829
M05.551	M05.812	M05.149	M05.69	M06.239	M05.10	M05.022
M05.449	M05.069	714.0	M05.542	M06.342	M05.142	M05.239
M06.051	714.1	M05.819	M05.412	M06.351	M05.461	M06.311
M06.832	359.6	M05.862	M06.252	M05.261	M06.031	M06.38
M05.042	714.89	M05.021	M05.00	M05.60	M05.351	M06.831
M05.352	714.9	M06.019	M05.262	M05.821	M05.49	M05.171
M05.669	M05.572	M06.219	M05.479	M06.00	M05.762	M05.559
M06.1	M05.751	M06.241	M06.059	M06.319	M05.349	M06.329
M05.861	M06.242	M06.312	M05.632	M06.379	M05.359	M05.041
M05.879	M06.30	M06.349	M05.119	M06.842	M05.419	M05.329
M05.031	M06.332	M06.9	M06.361	M05.249	M05.421	M05.471
M05.371	M06.811	M05.079	M05.052	M05.342	M05.761	M05.80
M05.549	M05.051	M05.222	M05.059	M05.50	M05.772	M05.379
M05.462	M05.20	M05.622	M05.729	M06.039	M05.321	M05.422
M05.229	M05.251	M05.411	M05.811	M06.812	M05.739	M06.372
M05.441	M05.552	M05.442	M06.879	M06.821	M05.841	M05.011
M06.261	M05.731	M05.859	M05.221	M05.611	M06.222	M05.129
M06.271	M05.752	M06.341	M05.242	M05.651	M05.642	M05.139
M05.062	M05.779	M06.822	M05.269	M05.122	M05.132	M05.569
Viral Hepatitis						
O98.413	070.9	711.90	K72.00	B16.2	070.59	583.81
B18.9	357.1	711.60	B17.0	B19.0	357.3	711.80
K74.69	B16.1	582.81	B18.0	B17.2	070.43	070.22
B17.9	B18.1	O98.411	B20	B16.9	070.49	070.31
581.81	B19.9	B19.11	B19.20	B19.10	B18.2	070.53
070.42	070.21	K74.60	070.0	O98.419	Z00.5	G63
711.50	070.20	B17.8	070.6	580.81	070.23	O98.412
042	647.60	M01.X0	070.33	647.61	647.63	N08
570	V70.8	B17.10	711.70	357.4	070.32	B16.0
B18.8	B19.21	070.70	571.5	070.1	070.44	070.52
B17.11	B15.0	070.41	070.71	711.40	070.54	070.30

B15.9	070.51	..	..	..	..	..

© 2022 McCreary EK et al. *JAMA Network Open.*

eTable 3. Primary and Secondary Outcomes in an Unmatched Cohort of Patients Receiving Subcutaneous Monoclonal Antibody Treatment and an At-Risk Population of Patients Not Receiving Monoclonal Antibody Treatment

	No. Events, Event Rate (%)		Risk Ratio (RR) Estimates						
**28-Day Event Rate Outcomes**	**Treated**	**Non-treated**	**Unadj.**	**Adjusted by Propensity Score**			**Adjusted by IPW**		
**Casirivimab + Imdevimab and Nontreated**	**(n=665)**	**(n=3821)**	**RR**	**RR**	**(95% CI)**	**p-value**	**RR**	**(95% CI)**	**p-value**
Hospitalization or death	23 (3.5)	251 (6.6)	0.5	0.4	(0.3 – 0.7)	<.001	0.5	(0.4 – 0.6)	<.001
Hospitalization	23 (3.5)	208 (5.4)	0.6	0.5	(0.4 – 0.8)	.004	0.6	(0.5 – 0.7)	<.001
Death	1 (0.2)	80 (2.1)	0.1	0.1	(0.0 – 0.4)	.004	0.1	(0.0 – 0.1)	<.001
ED admission or hospitalization	40 (6.0)	376 (9.8)	0.6	0.6	(0.4 – 0.8)	<.001	0.6	(0.5 – 0.7)	<.001

Abbreviations: Unadj, unadjusted; IPW, Inverse Probability Weigh; CI, confidence interval; ED, emergency department.

© 2022 McCreary EK et al. *JAMA Network Open.*

eTable 4. Comparison of Characteristics in Subcutaneous mAb Treated Group and Nontreated Control Group

Characteristic	Unmatched			Matched		
	Treated	Nontreated		Treated	Nontreated	
	(N=969)	(N=4353)	p-value	(N=652)	(N=1304)	p-value
Age, mean (SD)	53.8 (16.7)	49.7 (21.5)	<.001	53.7 (16.9)	53.0 (19.3)	.44
Female gender, No. (%)	547 (56.4)	2542 (58.4)	.27	388 (59.5)	785 (60.2)	.77
Black race, No. (%)	49 (5.2)	407 (9.5)	<.001	35 (5.4)	50 (3.8)	.12
Corticosteroids as a home medication, No. (%)	240 (35.9)	1190 (30.9)	.01	231 (35.4)	448 (34.4)	.64
History of rheumatoid arthritis, No. (%)	24 (3.6)	62 (1.6)	<.001	20 (3.1)	30 (2.3)	.31
History of asthma, No. (%)	220 (32.9)	1395 (36.2)	.10	213 (32.7)	387 (29.7)	.18
History of obstructive sleep apnea, No. (%)	128 (19.2)	507 (13.2)	<.001	123 (18.9)	212 (16.3)	.15
History of hypertension, No. (%)	314 (47.0)	1422 (36.9)	<.001	303 (46.5)	591 (45.3)	.63
History of COPD, No. (%)	115 (17.2)	566 (14.7)	.09	108 (16.6)	202 (15.5)	.54
History of diabetes, No. (%)	112 (16.8)	559 (14.5)	.13	107 (16.4)	203 (15.6)	.63
History of solid organ or cell transplant, No. (%)	10 (1.5)	6 (0.2)	<.001	1 (0.2)	2 (0.2)	1.0
**Variables Not Included in Propensity Score**	--	--	--	--	--	--
History of smoking, No. (%)	227 (34.0)	777 (28.7)	.008	221 (33.9)	305 (33.5)	.88
Body mass index, mean (SD)	31.8 (7.5)	31.6 (7.7)	.47	32.0 (7.6)	32.1 (7.7)	.92
History of dyspnea, No. (%)	40 (6.0)	200 (5.2)	.40	40 (6.1)	75 (5.7)	.52
History of pulmonary hypertension, No. (%)	13 (1.9)	56 (1.4)	.34	11 (1.7)	21 (1.6)	.90
History of atrial fibrillation, No. (%)	33 (4.9)	167 (4.3)	.48	32 (4.9)	62 (4.7)	.88
History of valvular heart disease, No. (%)	31 (4.6)	178 (4.6)	.98	30 (4.6)	71 (5.4)	.43
History of coronary artery disease, No. (%)	73 (10.9)	320 (8.3)	.03	71 (10.9)	142 (10.9)	1.0
History of stroke, No. (%)	35 (5.2)	185 (4.8)	.63	33 (5.1)	84 (6.4)	.22
History of congestive heart failure, No. (%)	36 (5.4)	221 (5.7)	.72	32 (4.9)	85 (6.5)	.16
History of chronic kidney disease, No. (%)	34 (5.1)	185 (4.8)	.75	27 (4.1)	80 (6.1)	.07
History of fatty liver disease, No. (%)	28 (4.2)	106 (2.8)	.04	28 (4.3)	42 (3.2)	.23
History of cancer, No. (%)	70 (10.5)	310 (8.0)	.04	69 (10.6)	133 (10.2)	.79
History of chemotherapy, No. (%)	31 (4.6)	113 (2.9)	.02	28 (4.3)	37 (2.8)	.09

© 2022 McCreary EK et al. *JAMA Network Open.*

History of allergic rhinitis, No. (%)	78 (11.7)	517 (13.4)	.22	77 (11.8)	172 (13.2)	.39
History of viral hepatitis, No. (%)	12 (1.8)	44 (1.1)	.16	11 (1.7)	19 (1.5)	.70
ACE Inhibitors, No. (%)	118 (17.7)	525 (13.6)	.006	118 (18.1)	201 (15.4)	.13
Angiotensin II receptor blocker, No. (%)	69 (10.3)	316 (8.2)	.07	67 (10.3)	152 (11.7)	.36
Alpha blocker, No. (%)	9 (1.3)	44 (1.1)	.65	9 (1.4)	16 (1.2)	.78
Beta blockers, No. (%)	166 (24.8)	708 (18.4)	<.001	158 (24.2)	272 (20.9)	.09
Statins, No. (%)	223 (33.4)	1018 (26.4)	<.001	216 (33.1)	425 (32.6)	.81
Antidepressants, No. (%)	200 (29.9)	1115 (28.9)	.60	194 (29.7)	414 (31.7)	.37
Charlson Comorbidity Index Score, mean (SD)	0.8 (1.4)	0.7 (1.3)	.03	0.8 (1.4)	0.8 (1.4)	.96

Abbreviations: SD, standard deviation; COPD, chronic obstructive pulmonary disease; ACE, angiotensin -converting enzyme.

© 2022 McCreary EK et al. *JAMA Network Open.*

eTable 5. 28-Day Hospitalization Outcomes by Route of mAb Administration

Outcome	Subcutaneous No. (%)	Intravenous No. (%)	Absolute Risk Difference (%)		
**All Infused Patients[a]**	**(n=969)**	**(n=1216)**	**Unadj.**	**Adj.**	**95% CI**
Hospitalization	27 (2.8)	20 (1.6)	1.1	1.5	-0.3 to 3.4
ICU admission	3 (0.3)	3 (0.2)	0.1	0.7	-3.5 to 5.0
Ventilation	3 (0.3)	2 (0.2)	0.1	0.2	-5.8 to 5.5
	**(n=27)**	**(n=20)**	**Wilcoxon p-value**		
LOS of hospitalizations, med, (IQR)	4 (2,8)	3 (4, 6.5)	0.70		
**Same Site Infused Patients[b]**	**(n=721)**	**(n=441)**	**Unadj.**	**Adj.**	**95% CI**
Hospitalization	17 (2.4)	4 (0.9)	1.5	1.5	-0.4 to 3.7
ICU admission	3 (0.4)	1 (0.2)	0.2	0.2	-5.9 to 6.3
Ventilation	2 (0.3)	1 (0.2)	0.1	0.0	-8.7 to 8.6
	**(n=17)**	**(n=4)**	**Wilcoxon p-value**		
LOS of hospitalizations, med, (IQR)	6 (3,10)	4.5 (2, 6)	0.42		

Abbreviations: mAb, monoclonal antibodies; Unadj, unadjusted; Adj, adjusted; CI, confidence interval; ICU, intensive care unit; LOS, length of stay; med, median; IQR, interquartile range.
[a]Patients treated at all health system facilities; SQ patients were treated from July 20 – September 20, 2021; IV patients were treated from July 15- September 29, 2021.
[b]Patients treated at the same UPMC facilities; SQ patients were treated from September 9-29, 2021; IV patients were treated from July 15- September 29, 2021. Adj: Model adjusted for age, gender, and vaccination status.

eFigure 1. Distribution of Propensity Scores (X 100) Before and After Matching of Treated and Not Treated Patients

The shaded rectangles depict the interquartile range; the lower and upper ends of the vertical lines depict the 5th and 95th percentiles. P-values are from Wilcoxon tests.

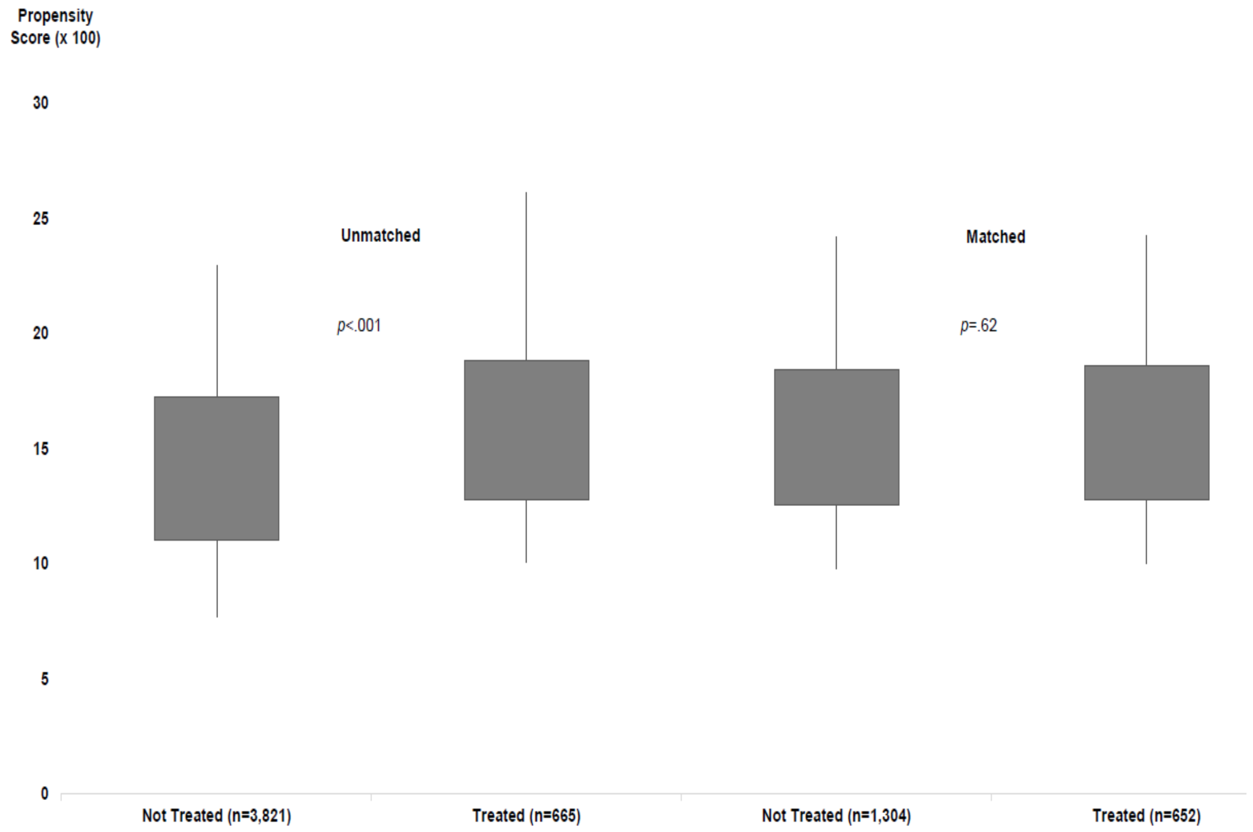

© 2022 McCreary EK et al. *JAMA Network Open.*

eFigure 2. Plot of Cumulative Event Rates of Hospitalization/Death by Day of Follow-up (X-Axis) for Matched Treated (Solid Line) and Not Treated (Dashed Line) Patients

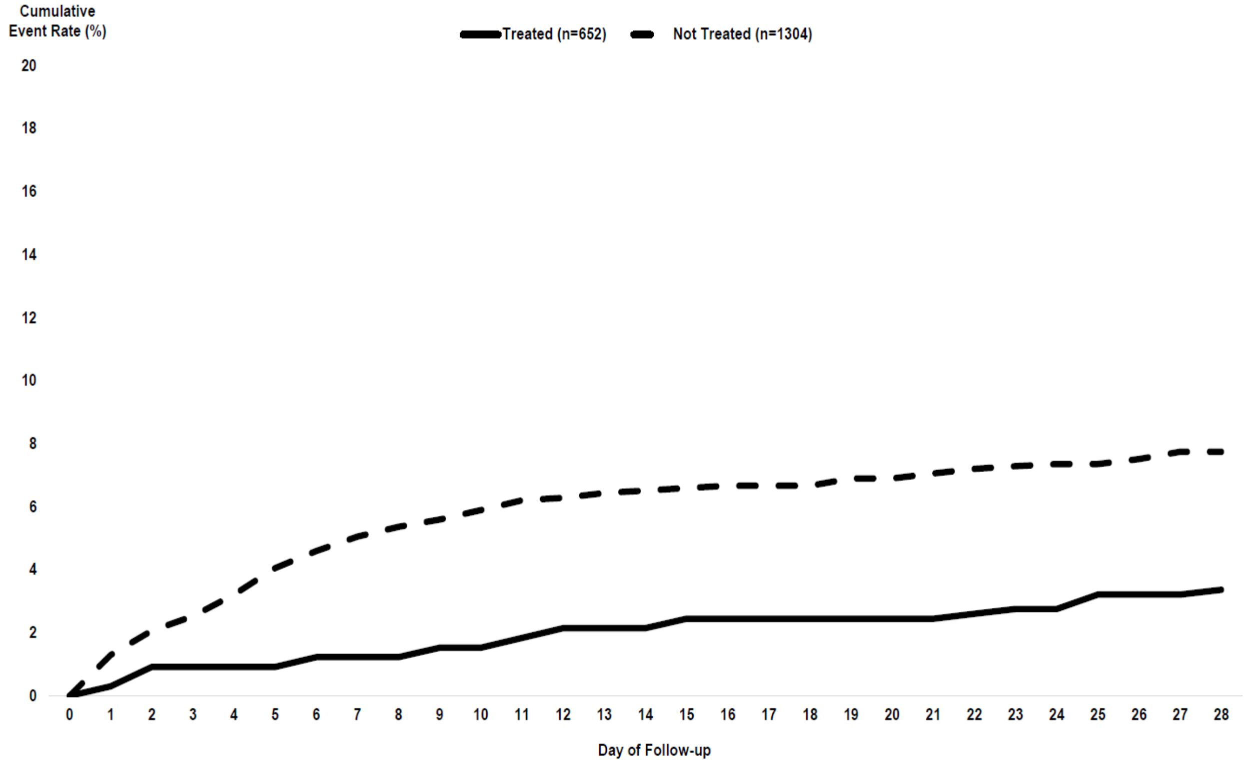

© 2022 McCreary EK et al. *JAMA Network Open.*
